# Synergistic Effects of Mechanical Properties and Self-Healing in Borate-Based Hydrogen-Bond Dynamic Crosslinked Gels in High-Temperature Water-Based Drilling Fluids

**DOI:** 10.3390/molecules31203675

**Published:** 2026-10-14

**Authors:** Leyao Du, Fan Zhang, Wenxiang Guo, Bo Jiang, Li Zhang, Xiaoli Ma, Liangbin Dou, Ziman Wei, Yongchao Wang, Jiahui Feng, Yongle Ren, Qi Li

**Affiliations:** 1 Engineering Research Center of Development and Management for Low to Ultra-Low Permeability Oil & Gas Reservoirs in West China (Xi’an Petroleum University), Ministry of Education, Xi’an 710065, China; duleyao0523@163.com (L.D.); 77129dou@163.com (L.D.); w3282378639@163.com (Z.W.); chaoswang0909@163.com (Y.W.); 17811106026@163.com (J.F.); 19992482529@163.com (Y.R.); 2 Shaanxi Key Laboratory of Advanced Stimulation Technology for Oil & Gas Reservoirs, Xi’an 710065, China; 3 Shaanxi Coal Geology Oil & Gas Drilling Co., Ltd., Xi’an 710048, China; shcfy131@126.com (W.G.); m18091591248@163.com (B.J.); zl_xsyu@163.com (L.Z.); m1536263323@163.com (X.M.)

**Keywords:** well leakage, self-healing gel, mechanical properties, leak-sealing effectiveness, leak-sealing mechanism

## Abstract

Engineering practices employ sealing materials such as rigid bridge plugs, cementation, and polymer gels to address leaking formations; however, each has limitations: particulate-based materials rely on a match between particle size and fracture width, while chemical cementation is constrained by temperature. Although polymer gels combine elasticity with filling capacity, conventional gels have a fixed crosslinked network prone to microcracking and volume loss under sudden load changes and high temperatures. This has prompted researchers to introduce self-healing gels with reversible crosslinking characteristics; yet, under extreme deep-well conditions, simultaneously achieving high mechanical strength, self-healing, and sufficient pressure-bearing capacity remains challenging. This study synthesized a self-healing gel through dynamic covalent crosslinking. High-temperature, high-pressure tests showed that a 5% gel achieved a burst pressure of 10 MPa at 120 °C. In cyclic tensile tests, the intact gel at 200% strain showed 37 kPa, whereas for gels lacking acrylamide or tannic acid components, the stress dropped to 5 kPa or lower, confirming that the crosslinked network confers excellent tensile properties and repeatable self-healing capability. In rheological experiments, after aging at 180 °C, the storage modulus (G’) of the gel remains at approximately 1000 Pa, maintaining its gel state and possessing high-temperature sealing potential. This gel offers a novel solution for sealing drilling leaks.

## 1. Introduction

One of the main elements limiting drilling efficiency, safety, and financial gains is wellbore leakage [1,2,3,4], a complicated downhole condition marked by the substantial loss of drilling fluid into the formation [5,6,7,8]. Injected into the leaking formation, gels [9] created by chemical bonding, intermolecular static interactions, and intermolecular forces create a sealing layer through filling and compression. Because of their superior flexibility, hardness, and filling capacity [10,11,12,13,14], they are utilized as traditional leak-sealing [15,16] materials. However, once fractured by shear or tensile forces, the crosslinked network of conventional gel plugging [17,18,19] materials is irreversible [20,21] and cannot self-heal [22] following damage; additionally, the plugging layer is vulnerable to crack propagation under repeated stress disturbances downhole, resulting in structural damage and plugging failure [23,24,25].

Self-healing gels [26], with their unique reversible crosslinking characteristics and damage self-healing properties [27,28], have been widely used as smart materials in fields such as biomedicine, flexible electronics, and coating protection [29,30,31,32]. Researchers both domestically and internationally have gradually applied them to drilling fluid leak control [33,34], addressing the shortcomings of conventional plugging gels. However, existing self-healing gels generally struggle to balance malleability, reusability, self-healing ability, and sealing capability in complex downhole conditions under high temperature and high pressure [35,36,37], while also exhibiting excellent tensile properties and rapid stress relaxation characteristics [38]. Although hydrophobic association-type gels exhibit high self-healing efficiency at room temperature, their mechanical strength decreases significantly [39,40]. For example, self-healing gels synthesized via micelle polymerization using acrylamide in combination with either methacrylate or acrylic esters in a sodium dodecyl sulfate-sodium chloride solution can achieve an elongation of up to 3600% at room temperature in air, and achieve 100% self-healing after fracture, but their tensile strength is only 10 kPa [41,42]. The hydrogen bonds in hydrogen-bonded gels can significantly enhance mechanical properties [43,44,45] through the synergistic interaction of multiple hydrogen bonds. For example, a self-healing gel exhibits an elongation at break as high as 118 times and a tensile strength of 175 kPa; however, its healing behavior is highly dependent on pH and temperature, making it highly prone to failure [46] under high-temperature conditions at the wellbore bottom. In terms of pressurized plugging, self-healing gels polymerized from monomers such as acrylamide (AM), acrylic acid (AA), and octadecyl methacrylate (SMA) exhibited a high energy storage modulus (3500 Pa) after self-healing at 90 °C and retained self-healing performance even at 120 °C. In a test using a 0.85 mm steel model to simulate a fracture, the leakage volume over 30 min at 120 °C and 3.5 MPa was 86 mL [47]. The P (AM-co-AMPS-co-LMA) hydrogel exhibited an elongation at break of 464.63%, with a recovery efficiency of over 87%. When cracks were simulated using 0.5 mm and 1 mm steel models, the hydrogel could withstand a pressure of 5 MPa only at 70 °C [48].

To resolve this contradiction, this study proposes a crosslinked network composed primarily of dynamic boronates generated by the reaction of polyvinyl alcohol (PVA) and boric acid (BA), into which acrylamide (AM) and tannic acid [49,50,51] (TA) have been incorporated. Through hydrogen-bond-assisted crosslinking formed between the amide groups of AM and the hydroxyl groups of PVA, as well as the phenolic hydroxyl groups of TA, this study synthesized a crosslinked network self-healing leak-sealing gel featuring dynamic borate ester covalent bonds and multiple hydrogen bonds. By leveraging the excellent high-temperature stability of borate ester bonds and the toughening effect of hydrogen bonds, this gel overcomes the mechanical strength limitations inherent in single dynamic covalent bond structures, enabling reversible crosslinking of gel particles within cracks and achieving complete sealing. Through experimental and theoretical analysis, this study (1) proposed a borate-hydrogen bond synergistic structure, resolving the conflict between mechanical performance and self-healing capability at high temperatures; (2) systematically elucidated the synergistic energy dissipation and self-healing mechanisms of the crosslinked network at high temperatures (180 °C), revealing the high-temperature reorganization characteristics of borate-ester bonds; (3) achieved high-temperature (120 °C) pressure-bearing sealing at 10 MPa in a 1 mm crack model, outperforming existing methods, and established a correlation between high-temperature rheological thresholds and sealing performance.

## 2. Results and Discussion

### 2.1. Chemical Structure of Gels and Their Self-Healing Mechanisms

#### 2.1.1. Fourier Transform Infrared Spectroscopy (FT-IR) Characterization

**Figure 1:**
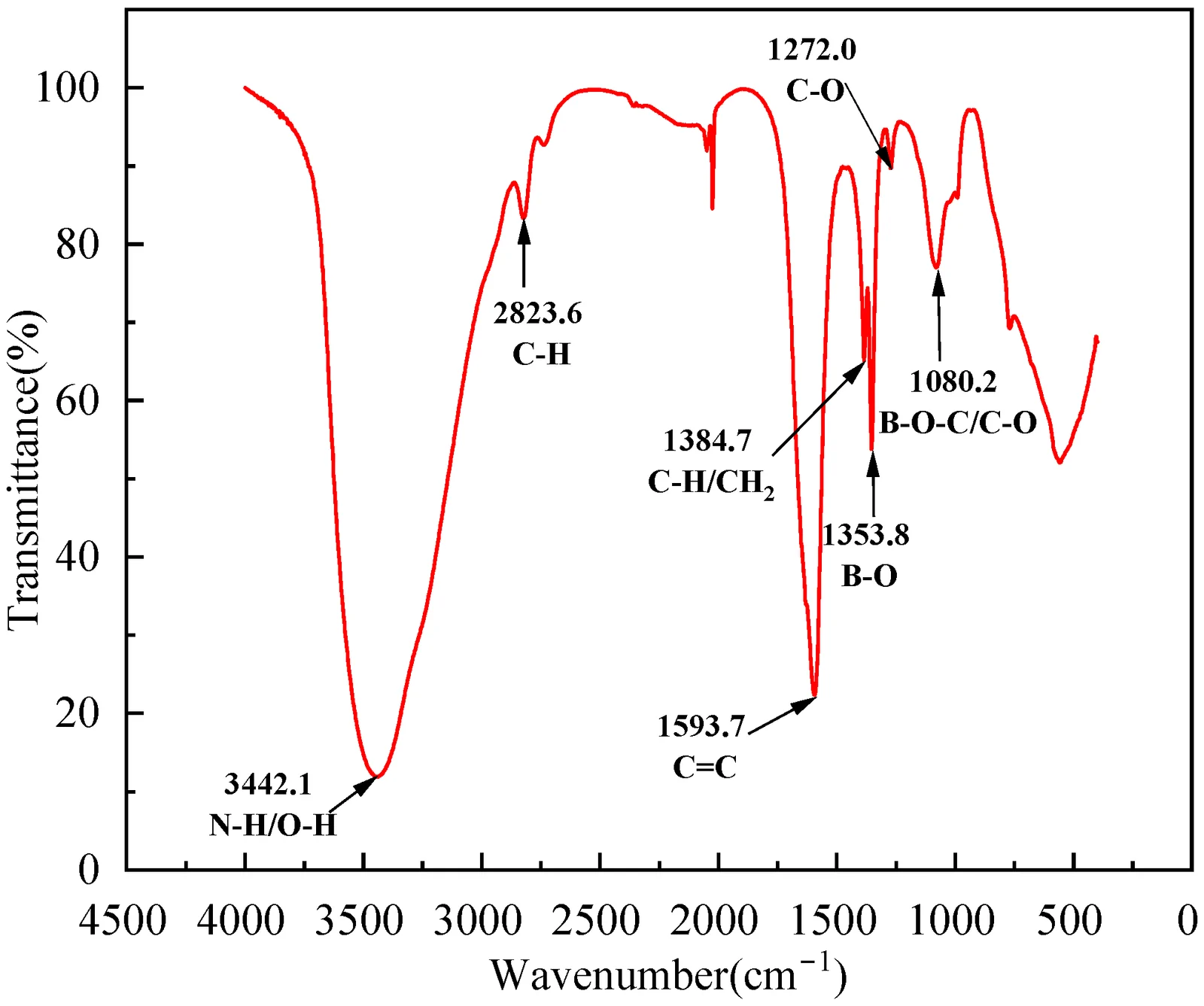
Infrared spectra of the gel.

As shown in Figure 1, Fourier transform infrared spectroscopy (FTIR) was used to characterize the chemical structure of the gel network. A broad and intense absorption band at 3442.1 cm^−1^ was attributed to the overlapping O–H and N–H stretching vibrations, indicating strong hydrogen-bonding interactions within the system. The absorption peak at 2823.6 cm^−1^ was assigned to C–H stretching vibrations. The strong absorption band at 1593.7 cm^−1^ was mainly attributed to the C=C skeletal stretching vibrations of aromatic rings. The absorption at 1384.7 cm^−1^ was attributed to the possible overlap of C–H/CH_2_ deformation vibrations and vibrations associated with boron-containing structures. A more pronounced adjacent absorption peak was observed at 1353.8 cm^−1^ and was assigned to B–O stretching vibrations. The relatively weak absorption feature at 1272.0 cm^−1^ was mainly associated with C–O stretching vibrations. The broad and intense absorption band at 1080.2 cm^−1^ was attributed to the combined contributions of C–O stretching vibrations in the PVA chains and B–O–C stretching vibrations of borate ester structures, consistent with the proposed mechanism involving the formation of dynamic boron-containing crosslinking structures mediated by boric acid.

More broadly, recent work on PVA-based composite films demonstrated that incorporating water-hyacinth-derived cellulose nanocrystals into PVA–chitosan films simultaneously modified the surface morphology and improved both hydrophilicity and tensile strength [52]. Although that material relies on nanofiller reinforcement rather than dynamic borate crosslinking, it illustrates that the distribution of hydroxyl-rich intermolecular interactions and the packing of PVA chains can jointly regulate interfacial wettability and mechanical integrity. In the present PVA/BA/AM/TA gel, dynamic borate ester bonds provide reversible network junctions, whereas hydrogen bonding associated with AM, TA, and PVA supplies secondary physical reinforcement and energy-dissipation sites.

#### 2.1.2. Thermal Stability (TGA) Characterization

**Figure 2:**
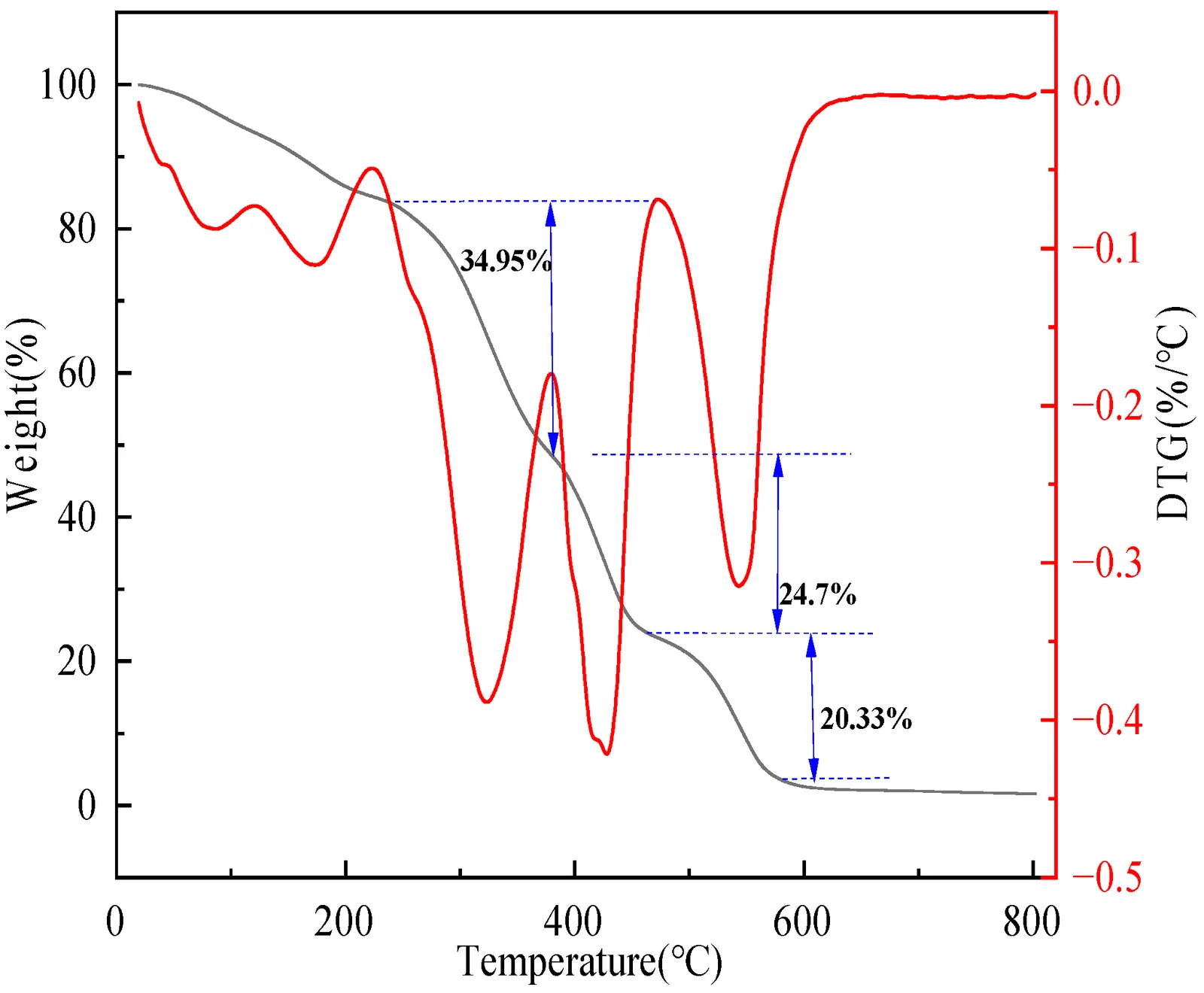
Thermal weight loss curves and thermal weight loss rate curves of the gel.

The thermogravimetric curve of the gel material is shown in Figure 2. The thermal degradation process of the sample can be divided into three stages: 240–380 °C, 380–463 °C, and 463–580 °C. In the first stage (240–380 °C), the rate of mass loss of the sample increased significantly, with a mass loss of 34.95% during this stage. As the sample had been washed with ethanol and dried at 60 °C prior to testing, most of the free water, weakly bound water, residual ethanol and soluble small molecules had been removed. Consequently, the 34.95 per cent mass loss in this range was primarily attributable to the removal of strongly bound water, the disruption of the hydrogen-bond network between hydrophilic functional groups, and the initial thermal decomposition of structural units with low thermal stability within the gel network. As the temperature rose, the hydrogen bonds and other physical interactions between the hydrophilic functional groups were disrupted, causing the gel network to gradually relax and promoting the further decomposition of side chains and local cross-linked structures. In the second stage (380–463 °C), the specimen continued to lose mass, with a cumulative mass loss of 24.70%, indicating that violent thermal cleavage of polymer side-chain chemical bonds occurred within this temperature range, involving the gradual breaking of weak bonds such as C–O and C–H; this was the primary temperature range responsible for the destruction of the gel’s side-chain structure. In the third stage (463–580 °C), the specimen continued to exhibit significant thermal weight loss, with a weight loss of 20.33%. In this temperature range, the C–C covalent bonds in the polymer main chain broke, leading to the destruction of the gel’s main structure. When the temperature exceeded 600 °C, the TG curve levelled off, the residual mass of the specimen remained essentially constant, and the thermal decomposition reaction ceased.

#### 2.1.3. X-Ray Photoelectron Spectroscopy (XPS) Characterization

**Figure 3:**
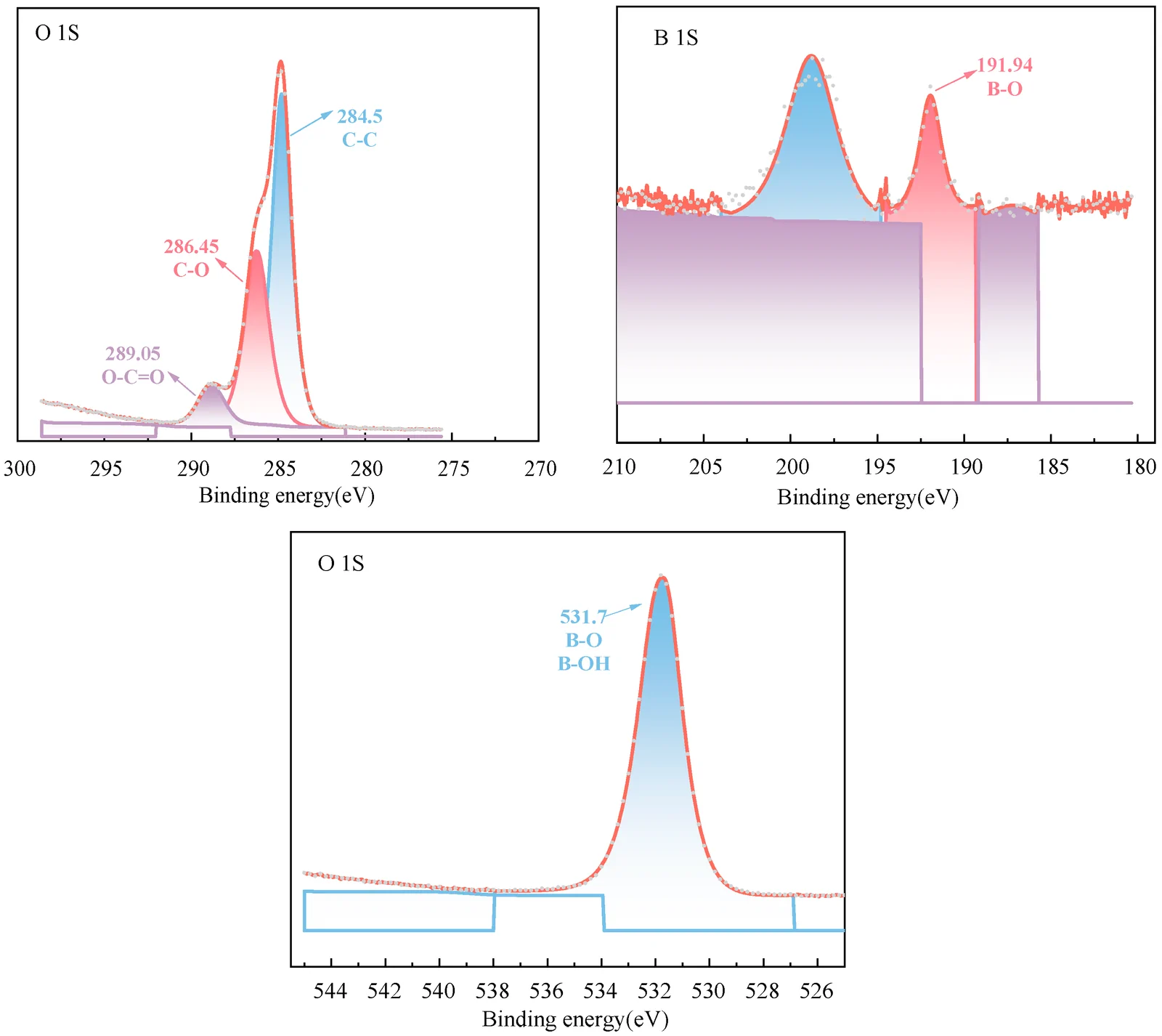
X-ray photoelectron spectroscopy (XPS) spectrum.

The chemical states of the system’s elements were analyzed using X-ray photoelectron spectroscopy (XPS, Thermo Kalpha, Waltham, MA, USA). Figure 3 depicts the C1s, B1s, and O1s spectra of the gel. The C1s spectrum, using the C–C bond at 284.3 eV as a reference, was resolved into three peaks: the peak at 284.3 eV corresponds to the saturated carbon backbone in the PVA main chain and acrylamide; the peak at 286.45 eV corresponds to the C–O bond; and the peak at 289.1 eV corresponds to the O-C=O groups formed in the gel system. The distinctive peak can be seen in the B1s spectrum: the B–O bond at 191.94 eV is mainly caused by the hydrolysis of boric acid and the creation of boron-oxygen coordination structures with tannic acid and PVA. B–O–C bond formation is shown by the double peak at 286.45 eV and 191.94 eV in the analysis above. Furthermore, Figure O1s shows a characteristic peak at 533.7 eV, which can be attributed to the B–O or B–OH bond. These XPS functional groups of the constituent polymers. Taken together, the results support a network in which dynamic borate junctions provide reversible crosslinks, while multiple hydrogen-bonding interactions enhance network cohesion and energy dissipation.

### 2.2. Evaluation of the Effect of Gel Particles on the Rheological Properties of Water-Based Drilling Fluids

Self-healing gel particles were added to the base fluid to evaluate the effects of the self-healing gel leak-sealing agent on the rheological properties and filtrate loss characteristics of the base fluid. To assess the gel’s impact on the rheological properties of the water-based drilling fluid, we measured the apparent viscosity (AV), plastic viscosity (PV), yield point (YP), and filtrate loss (FL_API_) at different gel concentrations.

**Figure 4:**
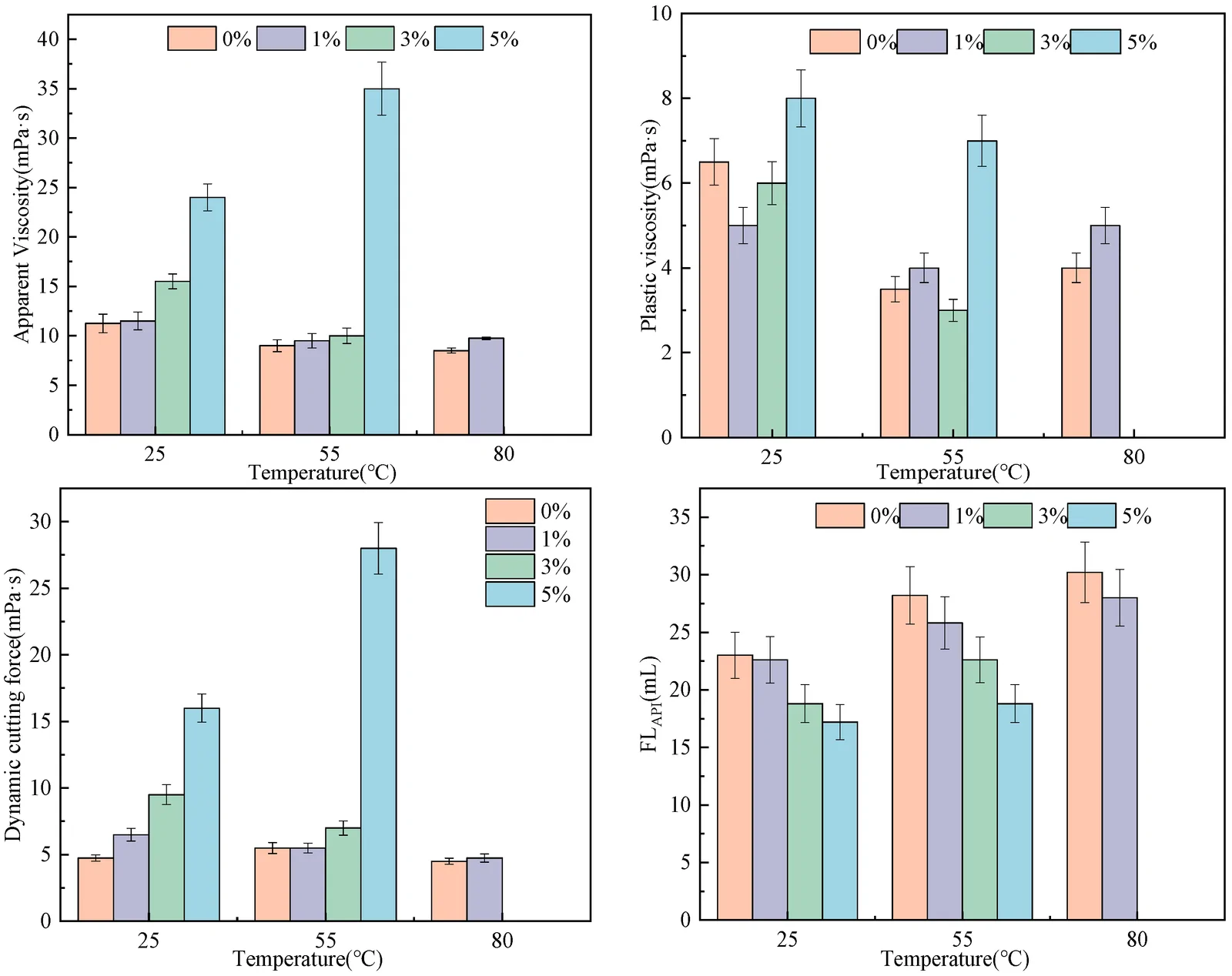
Performance testing of gel particles in water-based drilling fluids.

As shown in Figure 4, the experimental results indicate that the introduction of gel particles affects the rheological properties and filtration characteristics of the leak-sealing slurry. At 25 °C, as the gel concentration increased from 0% to 5%, the apparent viscosity (AV) rose from 11.25 mPa·s to 24 mPa·s, the yield point (YP) increased from 4.75 Pa to 16 Pa, and the API filtrate loss (FL_API_) decreased from 23.0 mL to 17.2 mL. This is attributed to the gel particles forming a structure through the synergistic action of dynamic reorganization of borate ester bonds and hydrogen bonding, which effectively enhances the gel’s colloidal suspension and cuttings-carrying capacity. At the same time, the dense structure improves the quality of the mud cake and enhances plugging performance. The gel exhibits good temperature adaptability: at 55 °C and 80 °C, AV and YP still show an upward trend with increasing gel dosage; and at 55 °C with a 5% gel addition, AV and YP reached 35 mPa·s and 28 Pa, respectively, indicating that dynamic borate ester bonds can maintain structural integrity at high temperatures, promoting the restructuring and strengthening of the gel-clay composite structure, thereby enabling the plugging slurry to maintain excellent resistance to shear-induced dilution. Furthermore, thanks to their self-healing capability, the gel particles can effectively fill the pores in the mud cake, significantly reducing fluid loss (e.g., at 25 °C and a 5% gel addition, FL_API_ decreased from 23.0 mL to 17.2 mL); however, upon heating to 80 °C, although the physical filling effect of the particles persists, the adsorption force between the particles and the rock surface weakens, reducing pore-filling capacity and consequently leading to increased fluid loss.

### 2.3. Study on Gel Wettability

Wetting theory is the core theory for studying the spreading ability of liquids on solid surfaces. Specifically, it examines the ability of various immiscible liquids to initially wet a solid surface when they come into contact with it. The contact angle is the most direct indicator for quantifying wetting behavior. To evaluate how the surface wetting characteristics of gels at different crosslinking densities affect their crack-sealing performance, and to reveal the structure–property relationship between the internal crosslinked network of the gel and its interfacial behavior, this study prepared thin films of gel solutions at concentrations of 5%, 7%, and 10%, and measured the static contact angle of deionized water on the film surfaces. For each concentration, measurements were taken at three points, with 40 readings recorded at each point.

**Figure 5:**
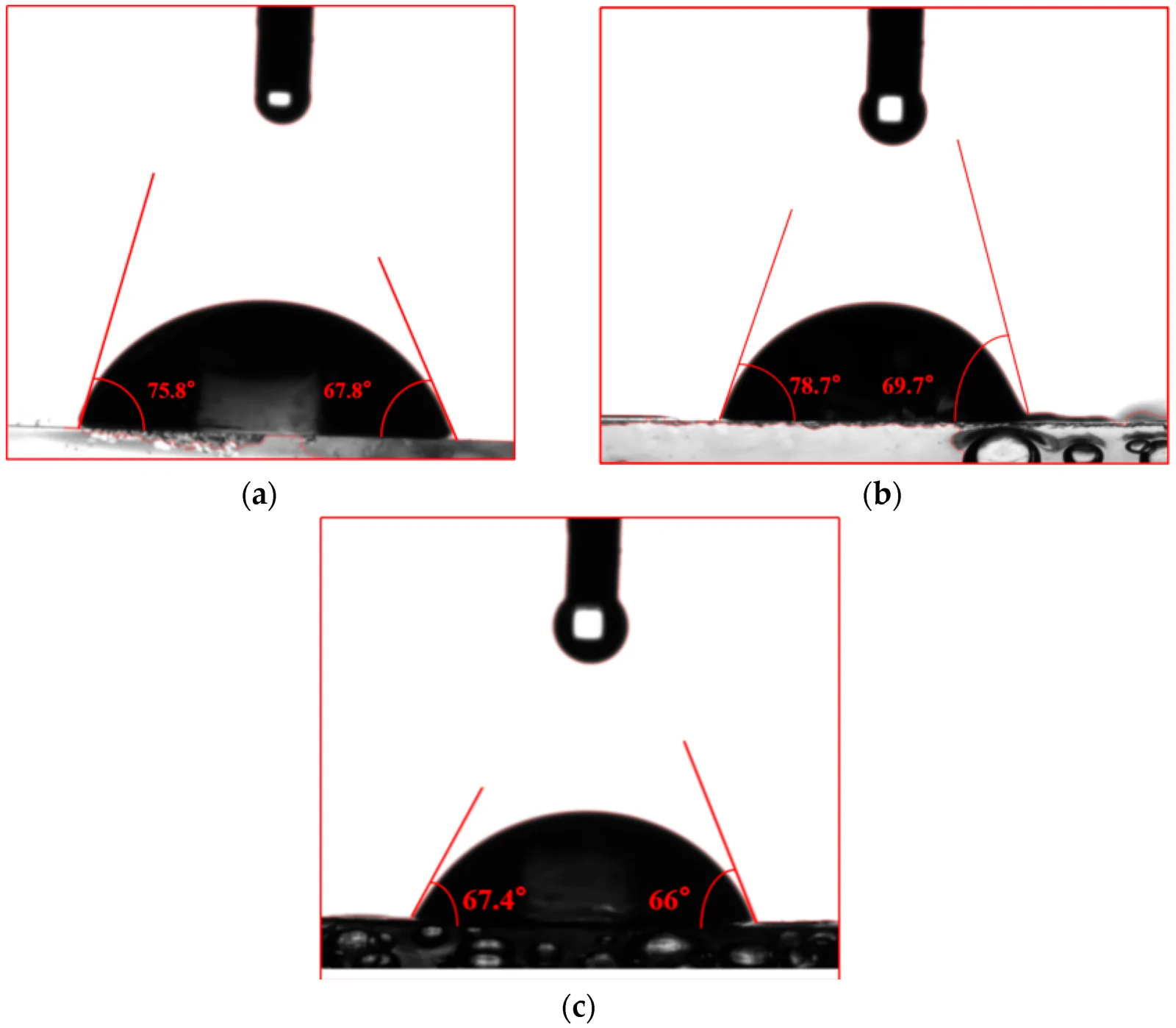
Static contact angles at different gel concentration. (**a**) Static contact angle at a 5% concentration; (**b**) Static contact angle at a 7% concentration; (**c**) Static contact angle at a 10% concentration.

As shown in the contact angle experiments in Figure 5 and Table 1, the self-healing gel exhibits weak hydrophilicity. The average contact angle of the 7% gel reached 71.30°, indicating a weakly hydrophilic surface. This is attributed to the moderate crosslinking density of the dynamic borate bonds formed between polyvinyl alcohol (PVA) and boric acid at this concentration. Furthermore, the gel structure at this concentration is relatively dense, which reduces surface hydrophilicity and increases the contact angle of water droplets. At the same time, in a hydrophilic rock fracture–water phase environment, reduced hydrophilicity facilitates the aggregation and spreading of gel particles on the hydrophilic rock fracture surfaces, reduces the repulsive potential at the solid–liquid interface, and promotes the retention of gel particles on the fracture walls while resisting erosion by formation water. Furthermore, the dynamic borate ester bonds on the PVA chains confer reversible crosslinking properties and self-healing capabilities to the gel; under pressure differentials, the gel undergoes further compaction and interfacial fusion, forming a continuous, dense sealing layer. At low concentrations, the density of borate ester crosslinking sites is insufficient, resulting in a loose gel structure with high porosity. The contact angle of the 5% gel is 62.23°, indicating relatively strong hydrophilicity; consequently, the integrity and pressure resistance of the sealing layer are limited. The contact angle of the 10% gel was 63.40°. Although the crosslinking density was further increased, excessively high concentrations may cause localized structural collapse or expose hydrophilic hydroxyl groups, thereby increasing hydrophilicity and reducing adhesion. The nonmonotonic concentration dependence indicates that surface wettability is not governed by nominal crosslink density alone, but reflects competition among the surface exposure of O–H/N–H groups, network compactness, and surface morphology. A comparable coupling between composition, interfacial properties, and mechanical reinforcement was reported for cellulose-nanocrystal-reinforced PVA–chitosan films, in which compositional modification simultaneously increased hydrophilicity and tensile strength and altered the surface morphology [53]. Therefore, a moderately crosslinking, weakly hydrophilic gel can achieve highly effective retention and sealing.

### 2.4. Analysis of the Mechanisms of Gel Self-Healing and Energy Dissipation

#### 2.4.1. Observation of Macro-Scale Self-Healing Behavior

**Figure 6:**
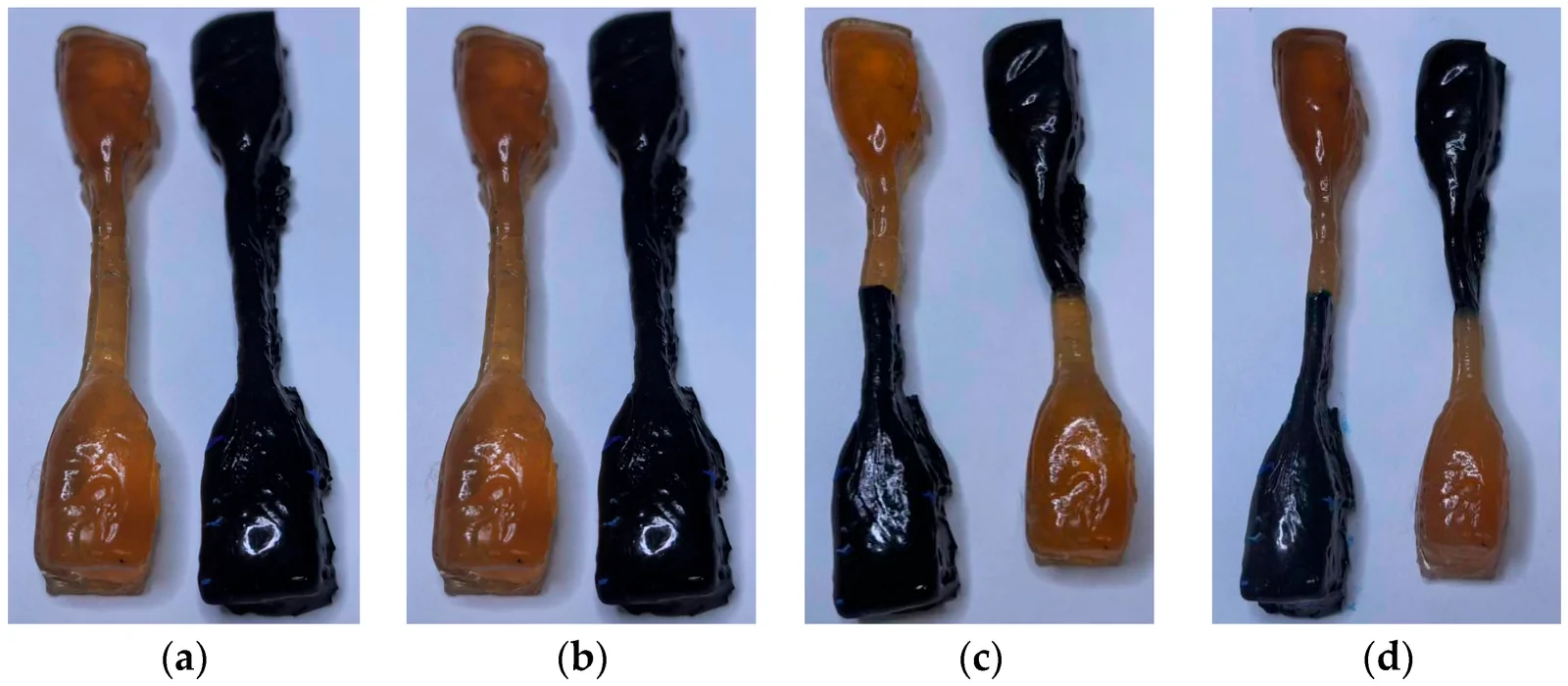
The self-healing process of the gel. (**a**) Before the incision; (**b**) After the incision; (**c**) Switch places; (**d**) Switch places.

As shown in Figure 6, the incisions of hydrogels of different colors healed completely in about 4 h. After 4 h of contact, the cracks disappeared, the incisions turned blue, and no white light was transmitted through. The two fracture surfaces had healed, achieving self-healing. This phenomenon is mainly attributed to the synergistic reconstruction of dynamic covalent bonds and non-covalent interactions in the hydrogel network: during the 4 h contact process, the borate bonds at the fracture surface undergo reversible breakage and reorganization in an aqueous environment. If the fracture surfaces remain separated for a long time, the surface moisture of the incision continues to evaporate, and the macromolecule molecular chains spontaneously undergo rearrangement. A large number of free hydroxyl and borate groups form intramolecular hydrogen bonds and borate bonds inside the fracture surfaces, significantly reducing the effective sites available for re-bonding at the interface, thereby hindering the dynamic bond reconstruction of the fracture surfaces and deteriorating the overall healing effect. This result indicates that in on-site construction, the time interval between the injection of gel particles and the closure of fractures should be minimized to ensure sufficient contact between the active sites of the fracture surfaces and fully utilize the self-healing ability of the gel.

#### 2.4.2. Observation of Microscopic Self-Healing Behavior

**Figure 7:**
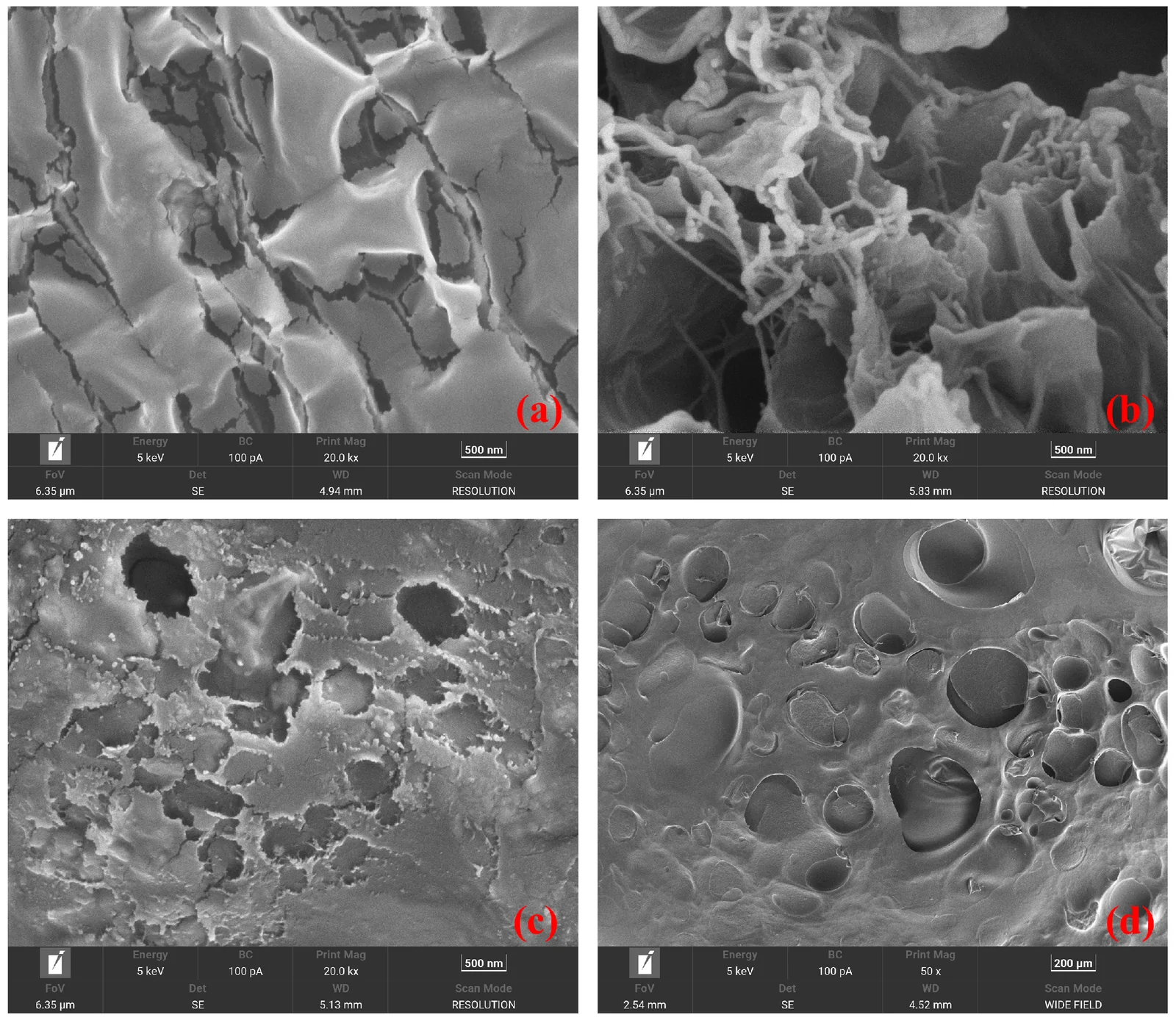
Scanning electron microscope (SEM) image: (**a**) Before gel incision; (**b**) After the gel incision has healed; (**c**) Gel without acrylamide (AM); (**d**) Gel lacking tannic acid (TA).

To further investigate the formation mechanism of the crosslinked network and its impact on self-healing performance, the microstructure of the gel was characterized using a scanning electron microscope (SEM, TESCAN GROUP, a.s., Brno, Czech Republic). The results are shown in Figure 7.

Figure 7a shows the microscopic morphology of the complete crosslinked gel formed by acrylamide (AM) and tannic acid (TA) prior to sectioning. The gel exhibits a typical three-dimensional, interconnected, porous network. The pore walls are relatively smooth, the pore size distribution is relatively uniform, and no obvious phase separation or structural collapse was observed. These results indicate that the introduction of AM and TA each enhances the formation of the hydrogen-bond, and the synergistic dynamic crosslinking network formed with borate esters through dynamic covalent bonds provides the material with good structural integrity.

Figure 7b depicts the microstructure of the same gel cross-section after self-healing is completed. During the self-healing process, macromolecule segments diffuse and reorganize at the fracture site, resulting in the virtual disappearance of the initial cut crack. A large number of entangled, uniformly distributed nanofibers were generated in situ at the fracture interface, resulting in a three-dimensional, interconnected nanoporous bridge network. The fibers are homogeneous in diameter and interwoven constantly, demonstrating that this crosslinked network gel has exceptional self-healing properties.

In contrast, Figure 7c shows the gel morphology when AM are absent. The results indicate that the gel exhibits large-area collapsed, flake-like aggregates or loose, granular accumulations, and the regular honeycomb pore structure disappears. This suggests that physical crosslinking relying solely on TA is insufficient to support a stable, uniform three-dimensional nanostructure, leading to severe phase separation within the material. As shown in Figure 7d, the gel lacking TA exhibits spherical or honeycomb-shaped voids with thick, relatively isolated pore walls. Due to the absence of hydrogen bonds provided by polyphenolic hydroxyl groups, the diffusion and rearrangement of molecular chains at fracture interfaces are impaired, resulting in a significant decrease in the gel’s self-healing ability. At the same time, the absence of TA causes the gel’s pore connectivity and structural uniformity to be significantly lower than those of a complete crosslinked network gel.

Consequently, these differences in dry morphology are more accurately described as pronounced structural heterogeneity, consistent with partial phase separation. Chitosan–alginate–carrageenan hydrogels also exhibit similar composition-dependent behaviour. Phuc [53] et al. kept the alginate and carrageenan contents constant whilst varying the chitosan content, and found that a moderate chitosan content produced the most uniform porous structure and the best mechanical properties. Insufficient chitosan leads to inadequate network reinforcement, whilst an excess of chitosan results in supersaturation, increased heterogeneity and a slight decline in mechanical properties. Although this system is primarily based on ionic complexation and hydrogen bonding, both studies indicate that structural integrity depends on an appropriate composition window: an insufficient amount of interacting components leaves an inadequately connected network, whilst an excess or imbalance of components promotes heterogeneity or phase separation.

#### 2.4.3. Energy Dissipation Mechanisms Revealed by Cyclic Tensile Testing

**Figure 8:**
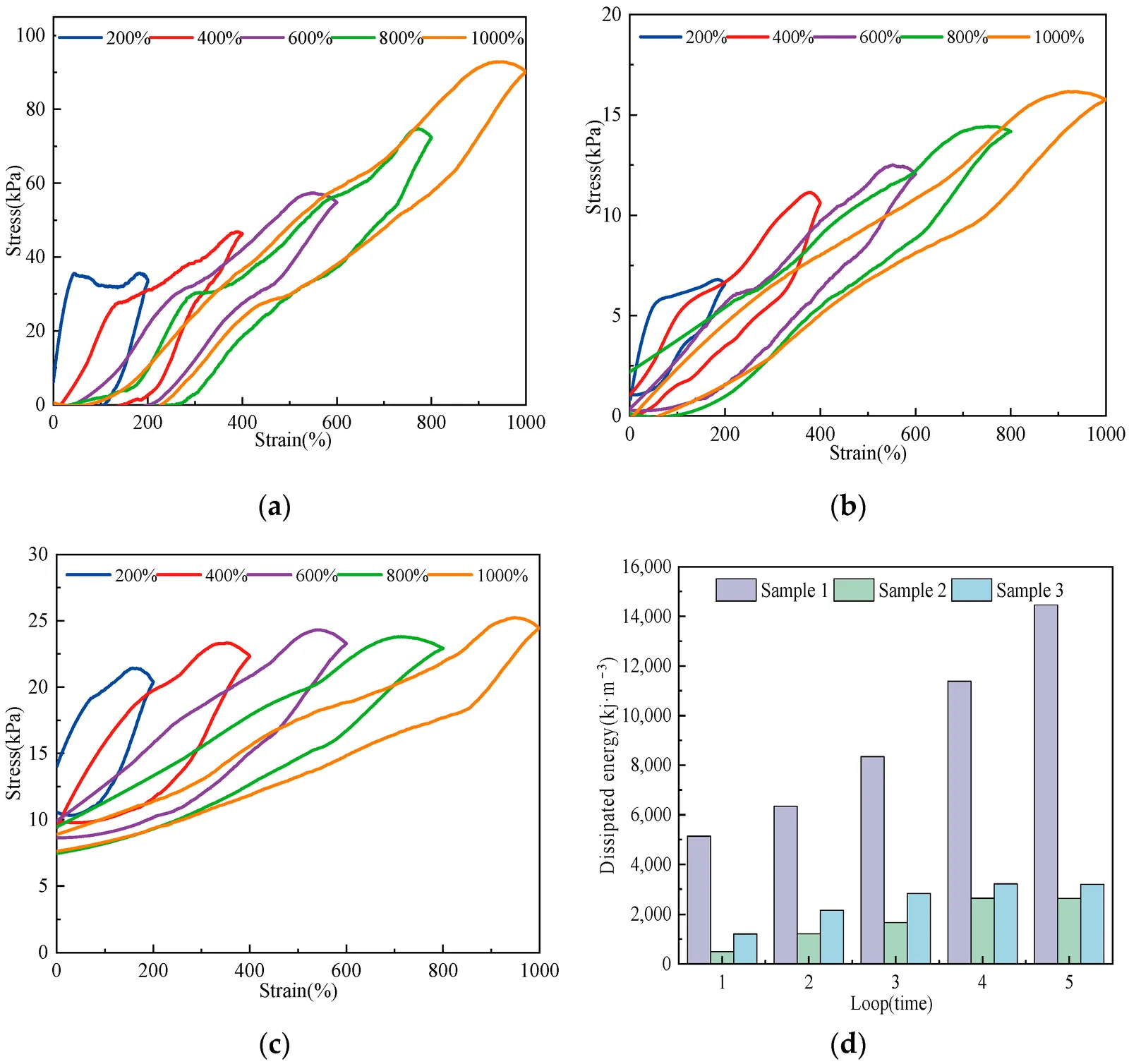
Cyclic tensile stress–strain curves and corresponding dissipated energy for the samples. (**a**) Cyclic tensile curve for Sample 1; (**b**) Tensile cycle curve for Sample 2; (**c**) Tensile cycle curve for Sample 3; (**d**) Energy dissipated during the cycle.

To investigate the effects of various components on the mechanical behavior of the gel and the mechanisms of energy dissipation, the fixed strain was set to 200%, 400%, 600%, 800%, and 1000%, and the results of the uninterrupted cyclic tensile testing of Samples 1, 2, and 3 were tested and compared, as shown in Figure 8. Sample 1 corresponds to the gel with all components intact; Sample 2 corresponds to the gel lacking acrylamide (AM); and Sample 3 corresponds to the gel lacking tannic acid (TA).

During the initial loading phase at 200% strain, the stress in Sample 1’s gel was approximately 37 kPa; as the strain increased, the stress required to reach the corresponding peak strain increased, indicating that the gel’s internal structure was gradually stretching and subjected to greater loads. Furthermore, after unloading to zero strain, all cycles showed separate closed hysteresis loops; as the number of cycles grew, so did the peak stress and loop area. This is due to the reversible breakdown of the polyvinyl alcohol-borate dynamic covalent connections and the dissociation/reformation of hydrogen bonds, which prevent the material from recovering promptly and completely during the unloading phase, resulting in stored energy. Sample 1 features a crosslinked network primarily composed of dynamic covalent borate bonds formed between polyvinyl alcohol and boric acid, supplemented by a physical network of hydrogen bonds to enhance stability. It exhibits both strong tensile properties and self-healing capabilities.

In a further comparison, Figure 8c shows Sample 3, whose mechanical properties and energy dissipation capacity have continued to decline compared to Sample 2. Although it retains a basic capacity for tensile deformation, both the peak stress and the area under the hysteresis loop have decreased significantly. This indicates that the absence of TA has further reduced the physical crosslinking density within the gel, particularly weakening the multiple hydrogen-bond interactions mediated by TA, thereby compromising the integrity of the structure and reducing its load-bearing capacity. Compared with Sample 2, Sample 3 exhibited lower stress responses and energy dissipation efficiency at all strain levels, confirming the key role of TA in constructing a highly physically reinforced network.

Strain is the percentage of elongation relative to the original length, whereas stress is the ratio of tensile force to original cross-sectional area. Toughness is defined as the area under the hydrogel’s stress–strain curve. The larger the area, the tougher the hydrogel sample. Energy is calculated by integrating the area under the hydrogel sample’s loading curve; stored energy is the area under the loading-unloading curve for a single cycle. As demonstrated in Figure 8d, the energy responses of the three samples under cyclic loading differ significantly. Sample 1 wasted considerably more energy per cycle phase than Samples 2 and 3. During the first cycle, Sample 1’s dissipated energy was 5141 kJ·m^−3^, which was approximately 10 times that of Sample 2 (494 kJ·m^−3^) and 4.25 times that of Sample 3 (1210 kJ·m^−3^); by the fifth cycle, it had further increased to 14,477 kJ·m^−3^. In contrast, Sample 2, due to the absence of AM, exhibits weakened intermolecular interactions, resulting in the lowest energy dissipation and a gradual increase; while Sample 3, lacking TA, although deficient in hydrogen bonds, could still provide basic viscoelastic energy dissipation through molecular chain reorganization, with its energy dissipation value falling between that of Sample 1 and Sample 2. In Sample 1, the synergistic effect of AM and TA enhances the energy dissipation efficiency associated with reversible fracture and microdamage accumulation, surpassing the superior energy dissipation performance observed when a single component is absent.

The correlation between SEM observations and tensile test results further supports the composition–structure–property relationship. In the chitosan–alginate–carrageenan system studied by Phuc et al., increasing the chitosan content from 0.50 g to 1.00 g raised the tensile strength from approximately 2.9 MPa to approximately 5.3 MPa and produced a more uniform porous morphology; however, a further increase led to a slight deterioration in mechanical properties and greater structural heterogeneity [53]. This comparison demonstrates that the mechanical enhancement of multicomponent hydrogels depends on the balance between cross-linking density, intermolecular compatibility and network uniformity.

As shown in Figure 8, the mechanical properties of the three groups of samples exhibit a decreasing trend. The introduction of AM and TA plays an indispensable role, at different levels, in the formation and reinforcement of the hydrogen-bonded physical network; furthermore, these components form an effective synergistic energy-dissipation mechanism with the dynamic covalent bonds of the boronates, jointly influencing the gel’s strength, tensile properties, and energy dissipation capacity.

### 2.5. Research on Leak-Stopping Performance Under High Temperature and High Pressure

A steel model with a diameter of 1 mm was used to simulate a highly permeable formation to evaluate the static plugging performance of the self-healing particle gel. As shown in Table 2, for Sample Solution 1, the downstream valve was opened the moment pressure was applied to the leak-sealing tester, causing the plugging slurry to flow out continuously; 115 mL flowed out within 10 min. After continuous pressurization, no further plugging slurry flowed out. After the experiment, the instrument was opened to reveal that no sealing slurry remained in the tank; it had been completely lost. For Sample Solution 2, as the pressure increased, there was zero leakage during the initial stage; the moment pressure was applied to the plugging tester, the downstream valve was opened, and a small amount of sealing slurry flowed out, totaling 5 mL within 10 min; upon continuous pressurization to 9 MPa, a cumulative total of 50 mL of sealing slurry flowed out within 10 min, demonstrating good sealing performance; pressure was further increased to 10 MPa, resulting in a cumulative outflow of 60 mL of sealing slurry within 10 min. As time elapsed, no additional sealing slurry continued to flow out, and the surface crack plate was effectively sealed. Therefore, the gel sealing layer is formed through the migration, aggregation, expansion, extrusion, and filling of gel particles within the cracks; the integral gel layer formed after healing possesses a higher load-bearing capacity.

**Figure 9:**
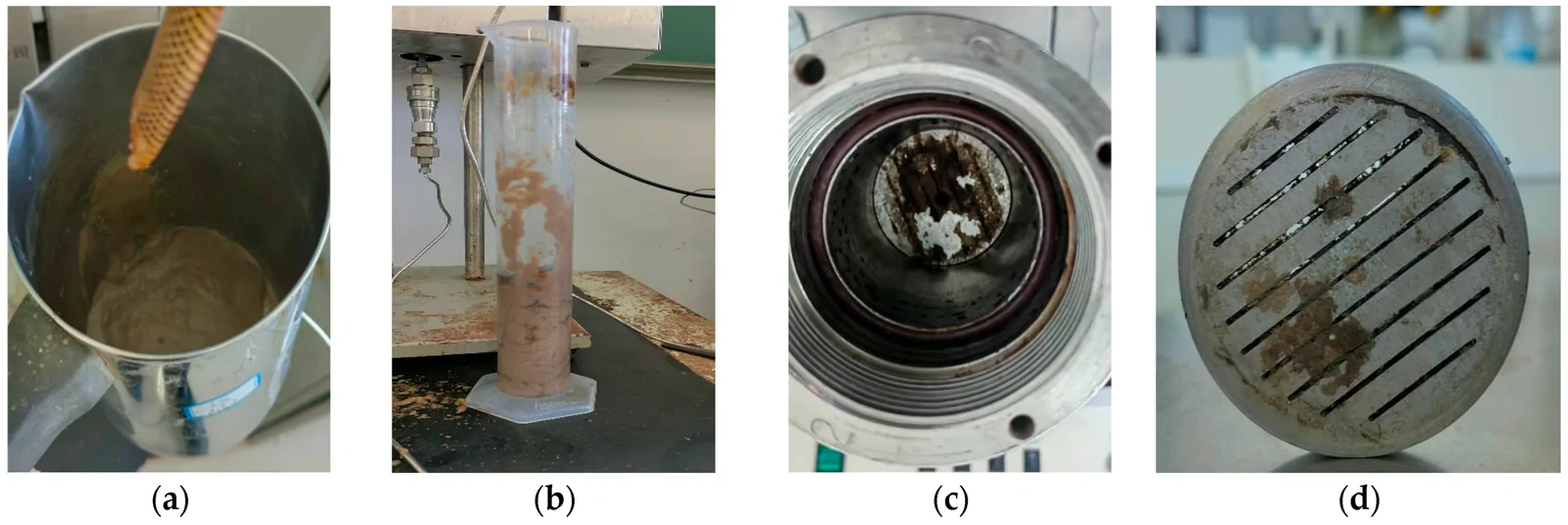
Observations from the grouting test (Sample Solution 1). (**a**) The grout used to seal the leak has begun to seep out; (**b**) Volume of grout lost during grouting; (**c**) Condition inside the tank after the experiment; (**d**) Sealing of the grout joint plate.

Figure 9a–c show the results of the experiment with Sample Solution 1. Figure 9d shows the condition of the slotted plate after being sealed with Sample Solution 1. After the experiment was completed, the slotted plate was removed, and it was found that the gaps had not been effectively filled, resulting in the loss of the grout.

**Figure 10:**
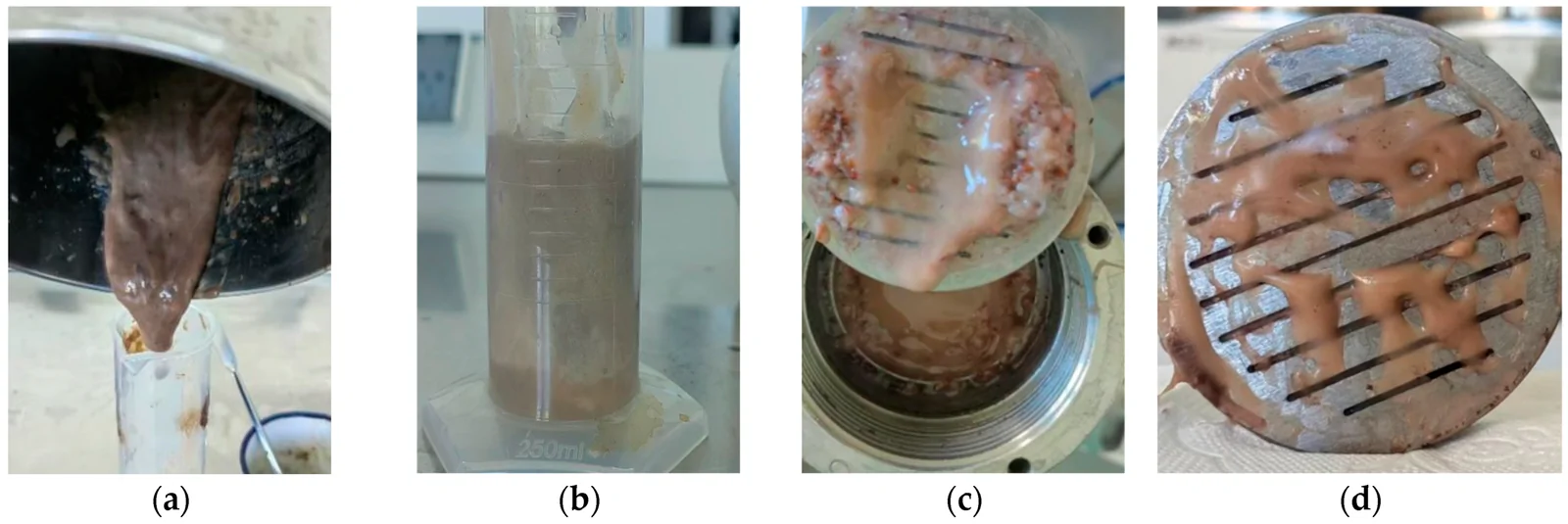
Observations from the grouting test (Sample Solution 2). (**a**) The grout used to seal the leak has begun to seep out; (**b**) Volume of grout lost during grouting; (**c**) Condition inside the tank after the experiment; (**d**) Sealing of the grout joint plate.

Figure 10a–c show the phenomena observed during the experiment with Sample Solution 2, while Figure 10d shows the sealing of the slotted plate in Sample Solution 2. After the experiment concluded, the reactor was opened, and the slotted plate was removed for inspection. It was found that all cracks had been effectively sealed, and the resulting sealing layer was dense, indicating that the bridging materials of different particle sizes had effectively served as bridges and fillers.

### 2.6. Rheomechanical Study

The storage modulus and loss modulus represent the energy stored and dissipated, respectively, when a material deforms under an external force. In dynamic rheology, the storage modulus G’ is a key parameter for measuring a material’s stiffness, while the loss modulus G” is related to the energy dissipated by the material during deformation. The storage modulus and loss modulus of a gel vary with changes in its structure over time and temperature. When the storage modulus is much greater than the loss modulus, the gel material exhibits overall solid-like properties; when the storage modulus is less than the loss modulus, the gel material exhibits overall liquid-like properties. In this paper, we use a rheometer to test the relationship between the loss modulus and the storage modulus to investigate the structural changes in self-healing gels under varying temperature and time conditions.

**Figure 11:**
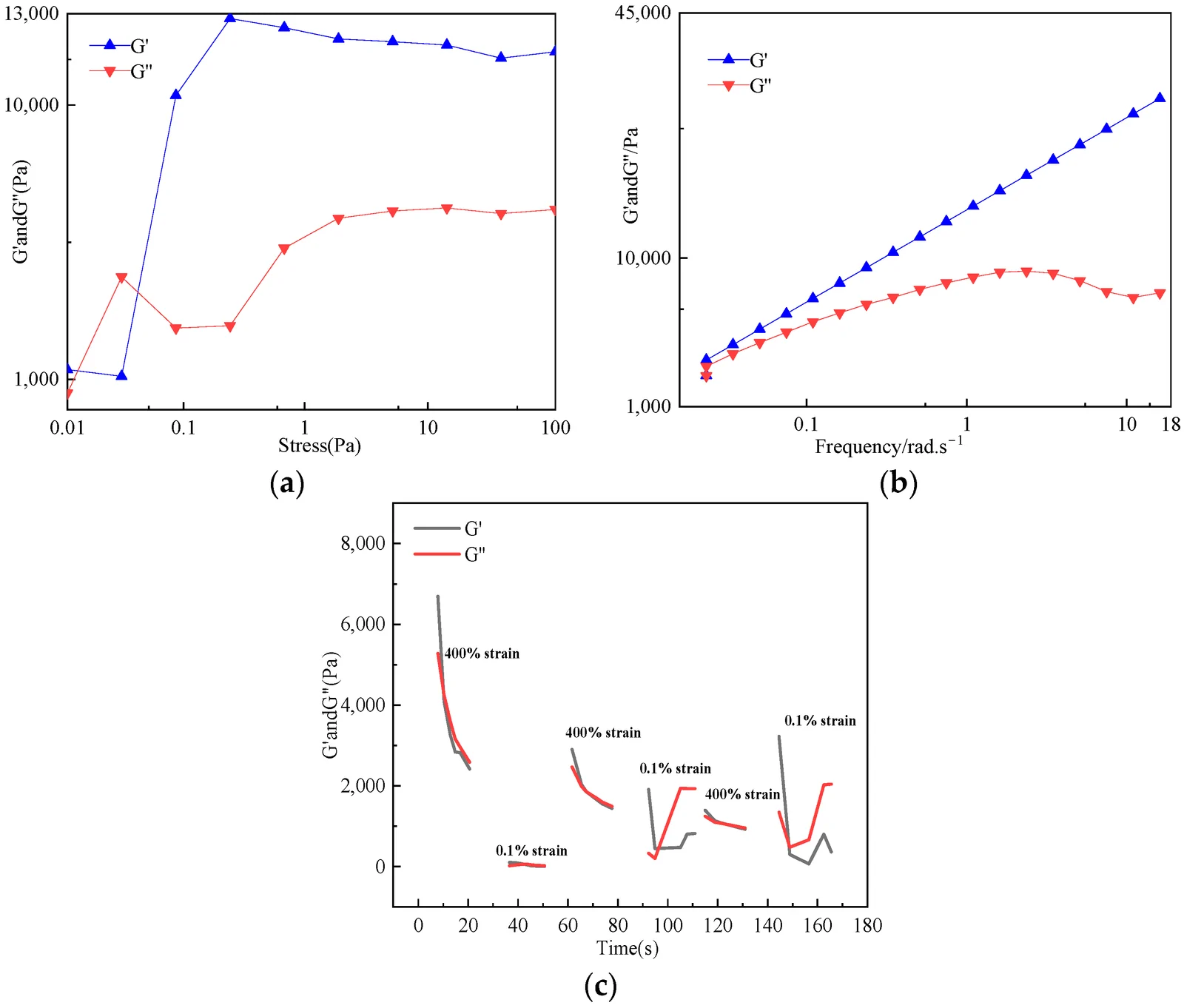
Rheomechanical properties of the gel at room temperature. (**a**) Variation of the gel’s storage modulus G’ and loss modulus G” with stress; (**b**) Variation of the gel’s storage modulus G’ and loss modulus G” with frequency; (**c**) Self-healing rheological properties of the gel.

At room temperature, the hydrogel exhibits the typical characteristics of an elastic solid. The frequency sweep results shown in Figure 11b indicate that, across the entire test frequency range, the storage modulus G’ (1000–30,000 Pa) consistently exceeds the loss modulus G” (1000–8000 Pa), and both increase approximately linearly with increasing angular frequency. This indicates that the hydrogel exhibits a predominantly elastic response under dynamic loading, with its energy storage capacity significantly exceeding its energy dissipation capacity, consistent with the typical characteristics of an elastic solid. Figure 11a demonstrates that during the stress sweep, G’ rises and reaches a peak (13,000 Pa) within the shear stress range of 0.01–0.1 Pa, while G” first increases and then decreases, indicating that the sample has entered the nonlinear viscoelastic region; G’ decreased slightly after reaching its peak and then leveled off when the stress ranged from 0.1 to 100 Pa, while G” rose generally and then slightly declined in the high-stress region before stabilizing. This suggests that hydrogen bonds continue to function as a buffer, allowing the gel to maintain its shear recovery capabilities even under severe deformation, while boronate covalent bonds break and reorganize under heavy shear.

Figure 11c, which shows step strain testing, confirms the gel’s repeatable self-healing capability. In the initial high-strain range of 400%, both the storage modulus G’ and the loss modulus G” dropped sharply (G’ decreased from approximately 6800 Pa to close to 2750 Pa, and G” decreased from approximately 5500 Pa to below 2750 Pa), but G’ remained greater than G”, indicating that the gel exhibited overall elastic behavior; this is primarily attributed to the reversible breaking of dynamic borate bonds within the gel, coupled with the gradual dissociation of the hydrogen bond, causing G’ and G” to decrease. When the strain returned to 0.1%, the modulus G” first dropped, then rose sharply and briefly exceeded G’, after which G’ gradually recovered. This reflects the reversible reformation of the broken dynamic borate covalent bonds, along with the gradual reestablishment of hydrogen bonds between molecular chains, endowing the gel with self-healing capability. Furthermore, the recovery trend observed during multiple cycles between 100 and 180 s in the figure indicates that the gel’s self-healing behavior is repeatable; however, repeated extreme deformations (multiple 400% strains) cause the peak value of G’ to gradually decrease (from 6800 Pa to 1800 Pa). This indicates that although the gel’s self-healing capability is cyclic, it remains limited by the energy changes associated with bond formation and dissociation; repeated extreme deformations result in irreversible degradation of mechanical properties.

Water content plays a significant role in the dynamic recovery process. An appropriate amount of absorbed water can plasticise the hydrophilic polymer chains, enhance segmental mobility, and facilitate the reversible re-formation of boronate bonds and hydrogen bonds once the external strain is removed. However, excessive swelling increases the interchain spacing, dilutes the cross-linking density, weakens intermolecular hydrogen bonds, and causes hydrolysis or rearrangement of borate esters, leading to a reduction in the number of effectively cross-linked elastic units and consequently a decrease in G′. Therefore, the maintenance of a quasi-solid response depends on a balance between sufficient hydration to provide molecular mobility and sufficient network connectivity to restrict chain escape and macroscopic dissolution. An example of a similar balance between water absorption and structural stability is found in chitosan–alginate–carrageenan composite films [54]. These films exhibit tensile strengths of 3.6–5.2 MPa and swelling ratios of up to 1347 per cent, whilst remaining insoluble in distilled water at pH 7. The results indicate that high water absorption does not necessarily lead to dissolution when ionic cross-links and interpolymer interactions maintain a continuous network.

**Figure 12:**
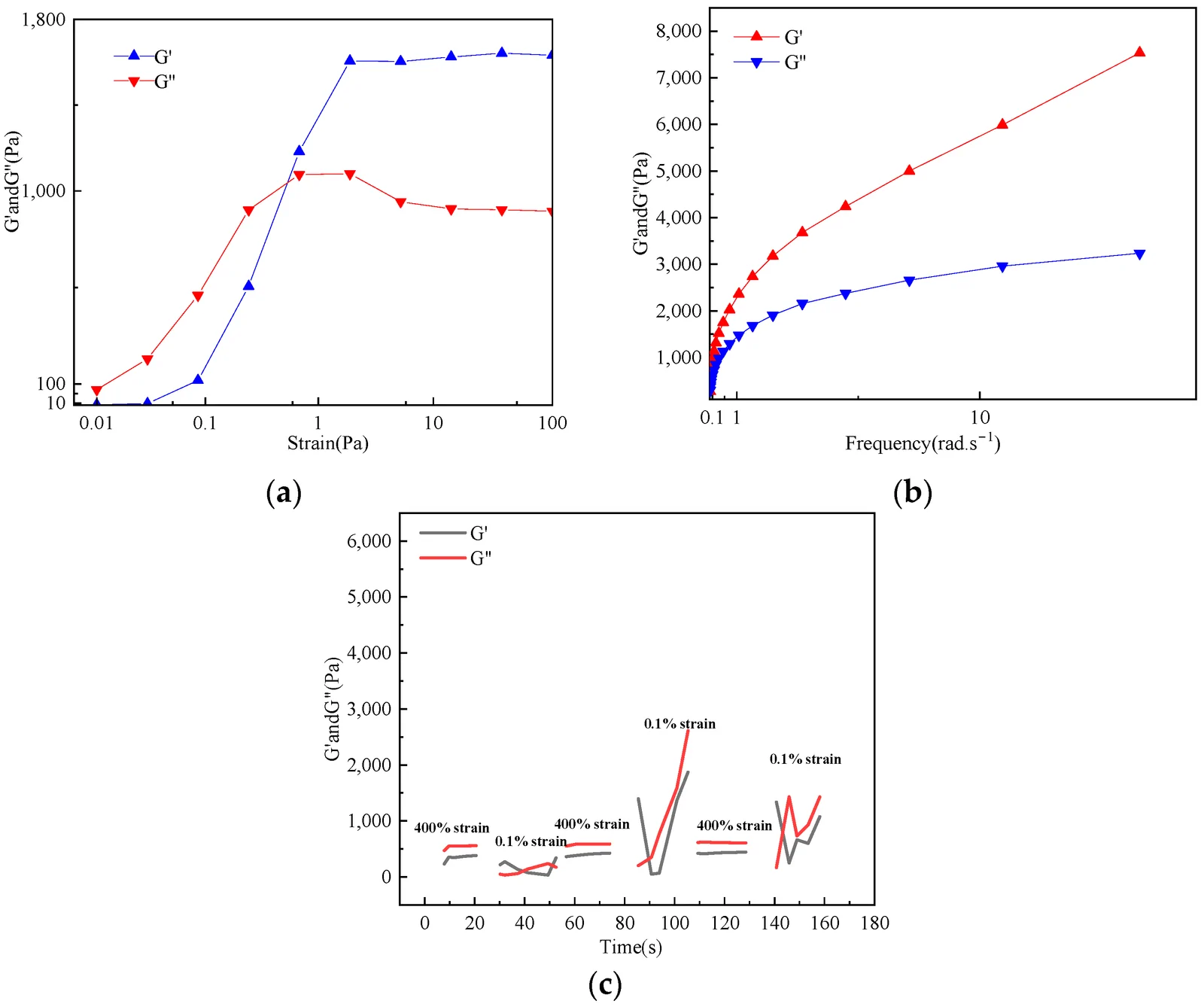
Rheomechanical properties of the gel at 150 °C. (**a**) Variation of the gel’s storage modulus G’ and loss modulus G” with stress; (**b**) Variation of the gel’s storage modulus G’ and loss modulus G” with frequency; (**c**) Self-healing rheological properties of the gel.

**Figure 13:**
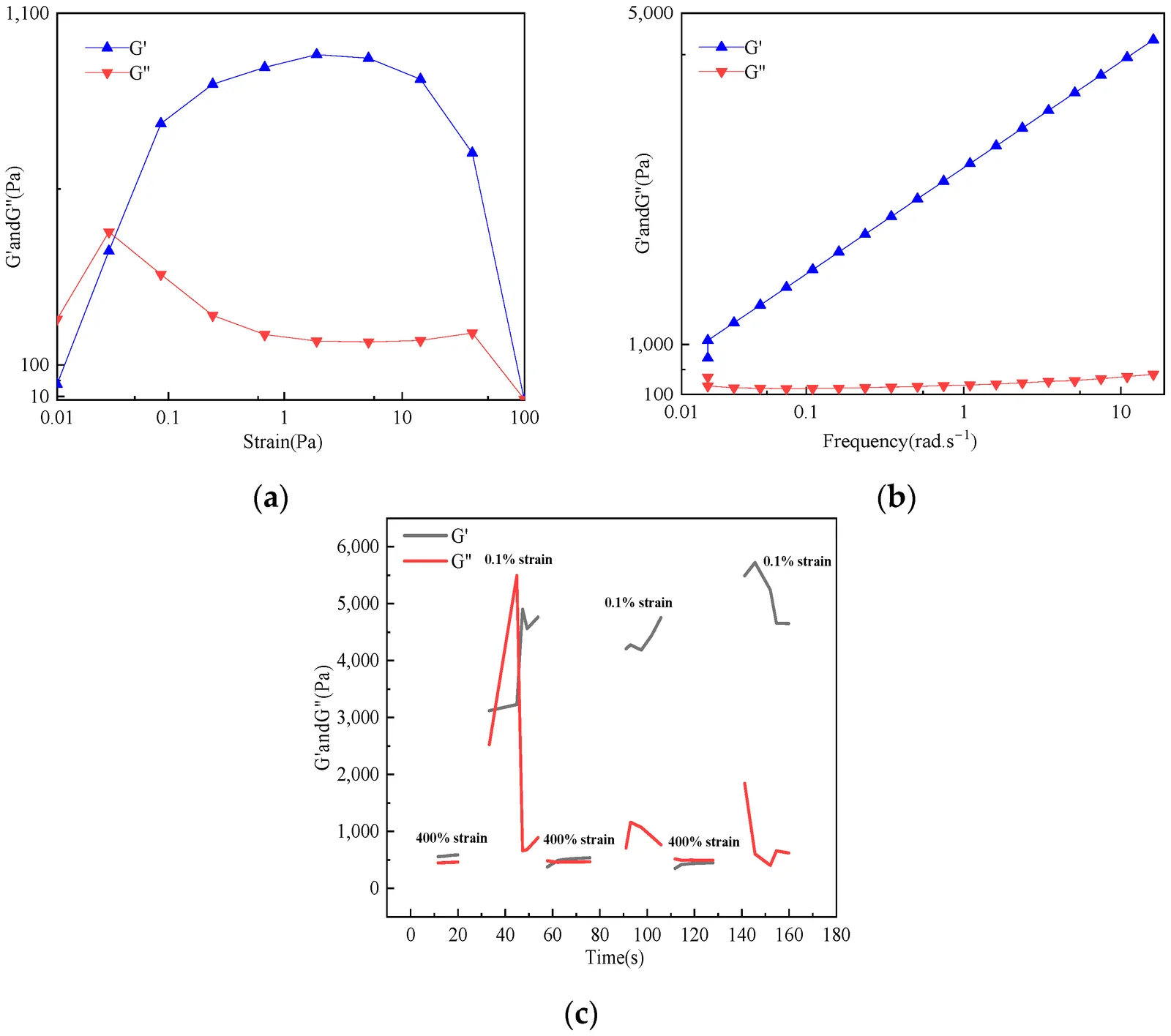
Rheomechanical properties of the gel at 180 °C. (**a**) Variation of the gel’s storage modulus G’ and loss modulus G” with stress; (**b**) Variation of the gel’s storage modulus G’ and loss modulus G” with frequency; (**c**) Self-healing rheological properties of the gel.

As demonstrated in Figure 12 and Figure 13, the gel’s mechanical strength declined during high-temperature aging, but the structure remained intact. After aging at 150 °C, the gel’s G’ peak reduced from roughly 11,000 Pa at ambient temperature to around 1000 Pa, and then to around 800 Pa at 180 °C. This is because high temperatures disrupted the intermolecular hydrogen bonds and boronate bonds, resulting in a decrease in crosslinking density, rigidity, and elasticity, causing mechanical strength to decline as temperature increased; however, G’ remained greater than G”, indicating that the gel framework was not completely destroyed and still retains its viscoelastic solid-state structure. Step-strain cycling tests show that, after high-temperature aging, the steady-state modulus of the gel decreases at a high strain of 400%, but when switched to a low strain of 0.1%, its structural recovery capability is superior to that of the gel at room temperature, and the modulus decays more slowly after multiple cycles.

**Figure 14:**
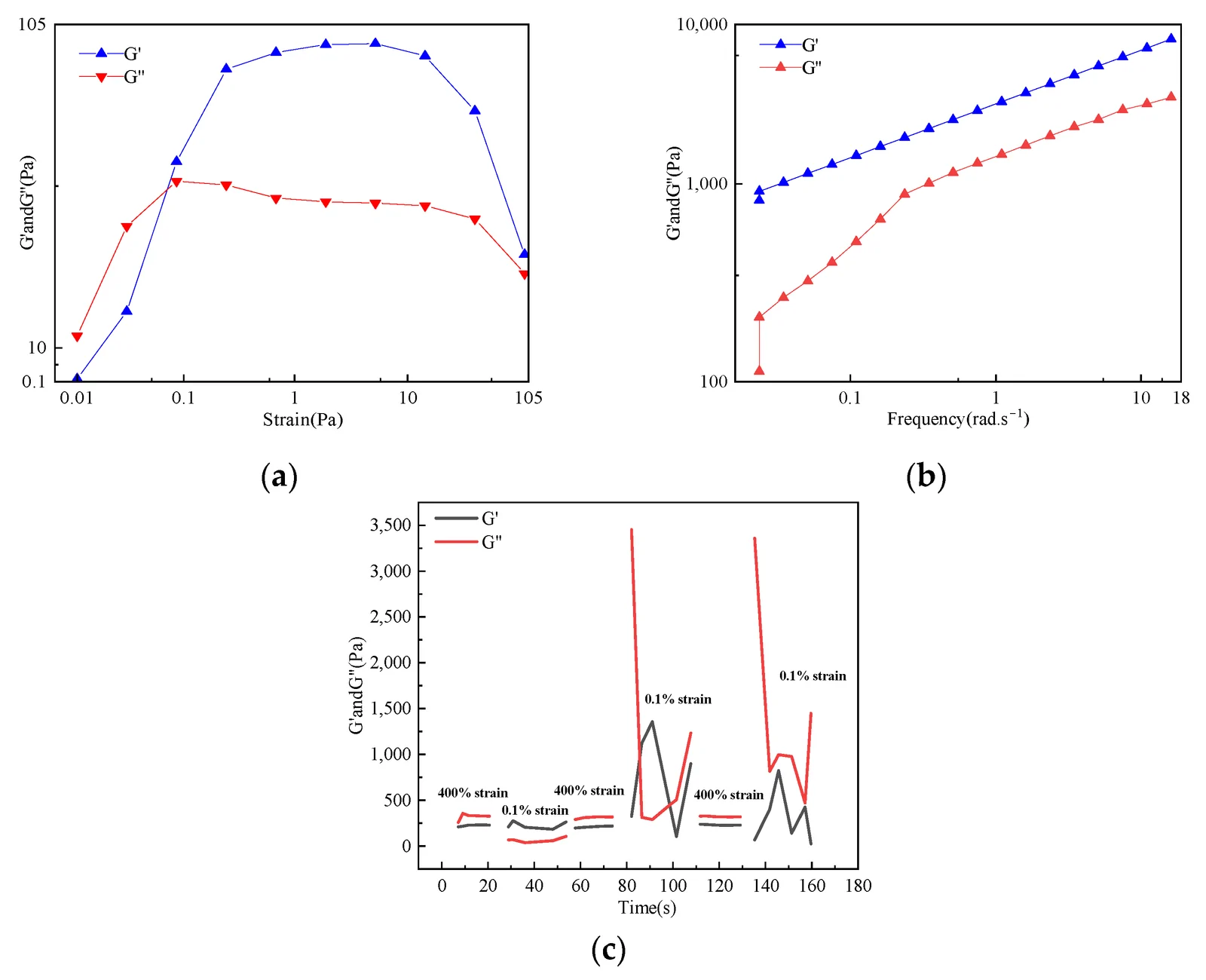
Rheomechanical properties of the gel at 150 °C. (3.5-h static aging phase) (**a**) Variation of the gel’s storage modulus G’ and loss modulus G” with stress; (**b**) Variation of the gel’s storage modulus G’ and loss modulus G” with frequency; (**c**) Self-healing rheological properties of the gel.

As shown in Figure 14, a 3.5-h static aging phase was created to replicate the gel’s performance evolution throughout its dwell time in downhole operations in order to fully examine the structural changes in the gel under limited subsurface settings. According to test results, the gel’s storage modulus (G’) and loss modulus (G”) generally showed a negative trend after 3.5 h of static aging, suggesting a decline in the crosslinked network’s dynamic shear resistance. However, static aging promotes further densification of the network, and this structural reorganization reduces the peak elastic recovery of the gel after a single large strain damage, but sacrifices some self-healing ability. Additionally, during deformation, the internal rearrangement caused by the stand increases energy dissipation. Although the self-healing recovery capacity is somewhat weakened after multiple cycles of failure, the gel still retains a high degree of elastic recovery, indicating that the material possesses good potential for cyclic self-healing.

**Figure 15:**
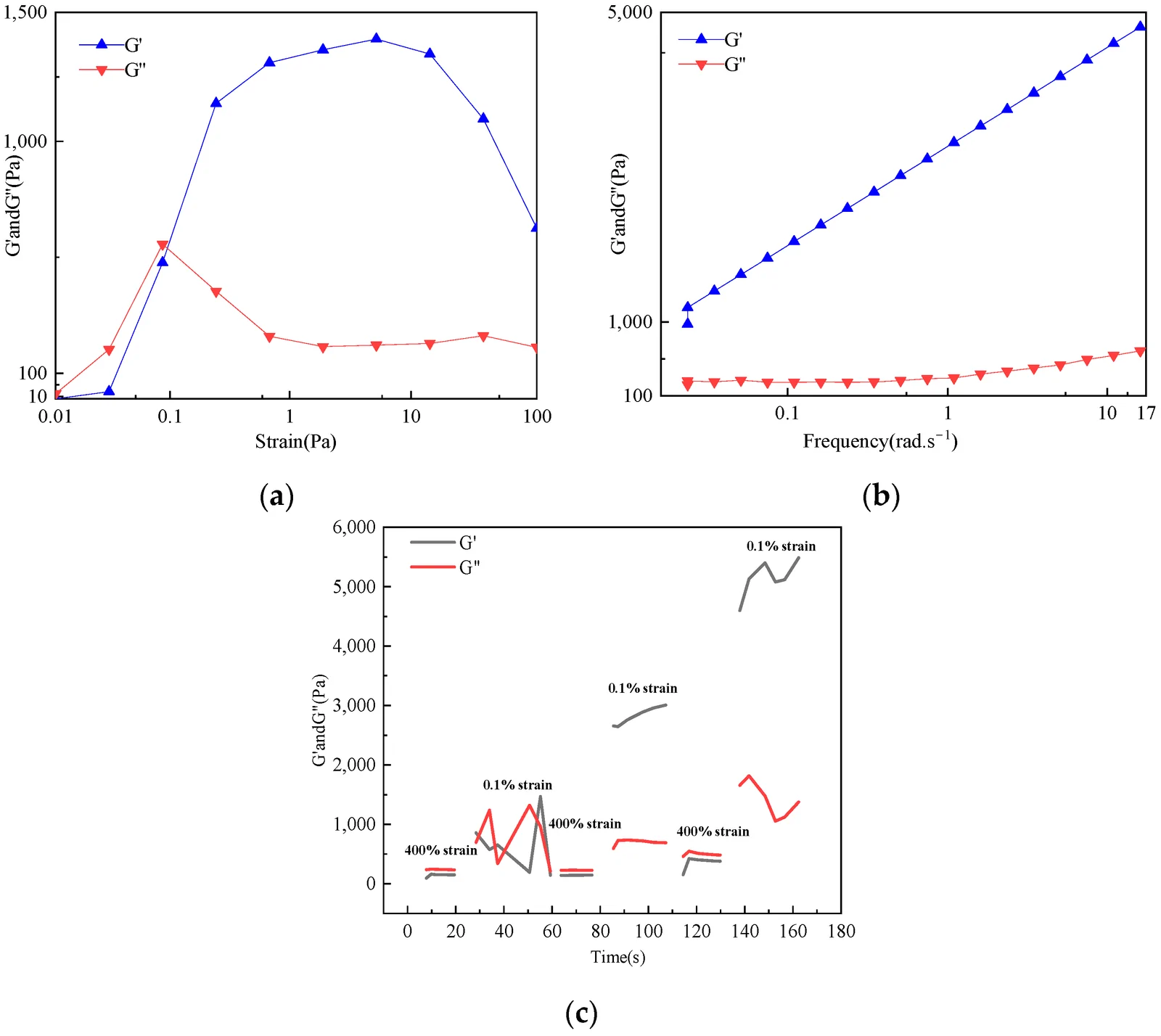
Rheomechanical properties of the gel at 180 °C. (3.5-h static aging phase) (**a**) Variation of the gel’s storage modulus G’ and loss modulus G” with stress; (**b**) Variation of the gel’s storage modulus G’ and loss modulus G” with frequency; (**c**) Self-healing rheological properties of the gel.

Figure 15 shows that the G’ of the gel remains at the 1000 Pa level after aging at 180 °C, and that G’ > G”, indicating that its solid-state structure has not collapsed.

## 3. Materials and Methods

### 3.1. Materials and Instruments

Polyvinyl alcohol (AR) was purchased from Shanghai Chenqi Chemical Technology Co., Ltd. (Shanghai, China); tannic acid (AR) and boric acid (AR) were purchased from Tianjin Aopusheng Chemical Co., Ltd. (Tianjin, China); acrylamide (AR) was purchased from Tianjin Damao Chemical Reagent Factory (Tianjin, China).

Heat-Collecting Constant-Temperature Magnetic Stirrer (DF-101S), Inverter-Controlled High-Temperature Roller Heater (GW300-X), Electric Six-Speed Viscometer (Model ZNN-D6, Qingdao Haitongda Special Instrument Co., Ltd., Qingdao, China), IRTracer-100 Infrared Spectrometer (Shimadzu Corporation, Tokyo, Japan), TGA-2 Thermogravimetric Analyzer (Mettler-Toledo, Shanghai, China), Medium-Pressure Filtration Loss Tester (Model SD-4, Qingdao Haitongda Special Instrument Co., Ltd., Qingdao, China), Electronic Universal Testing Machine (Xiamen Yishite Instrument Co., Ltd., Xiamen, China), Complex Fluid Flow Characteristics Tester (Haaker, Karlsruhe, Germany), High-Temperature, High-Pressure Static Leak Tester, X-ray Photoelectron Spectrometer (Thermo Kalpha, Waltham, MA, USA).

### 3.2. Preparation of the Gel

Using an electronic balance, accurately weigh out 12.65 g of polyvinyl alcohol (PVA) powder and 30 g of deionized water, and place them in a polytetrafluoroethylene (PTFE) container. Stir vigorously at 90 °C until the polyvinyl alcohol (PVA) is completely dissolved and a clear, homogeneous solution is formed. Dissolve 0.84 g of boric acid (BA) and 12.65 g of acrylamide (AM) in 16 mL of water to obtain mixed solution A. Dissolve 0.89 g of tannic acid (TA) in 4 mL of water to obtain mixed solution B. Add mixed solution A and then mixed solution B to the polyvinyl alcohol (PVA) solution in sequence. At this point, the weight ratio of PVA, BA, and TA is 15.06:1:1.06 (which yields optimal gel properties). Continue stirring; as the dynamic crosslinking reaction proceeds, the solution will gradually become extremely viscous. When the mixture becomes too viscous, switch to manual stirring and kneading with a metal spatula or glass rod for approximately 20–30 min, until a uniform gel forms.

**Figure 16:**
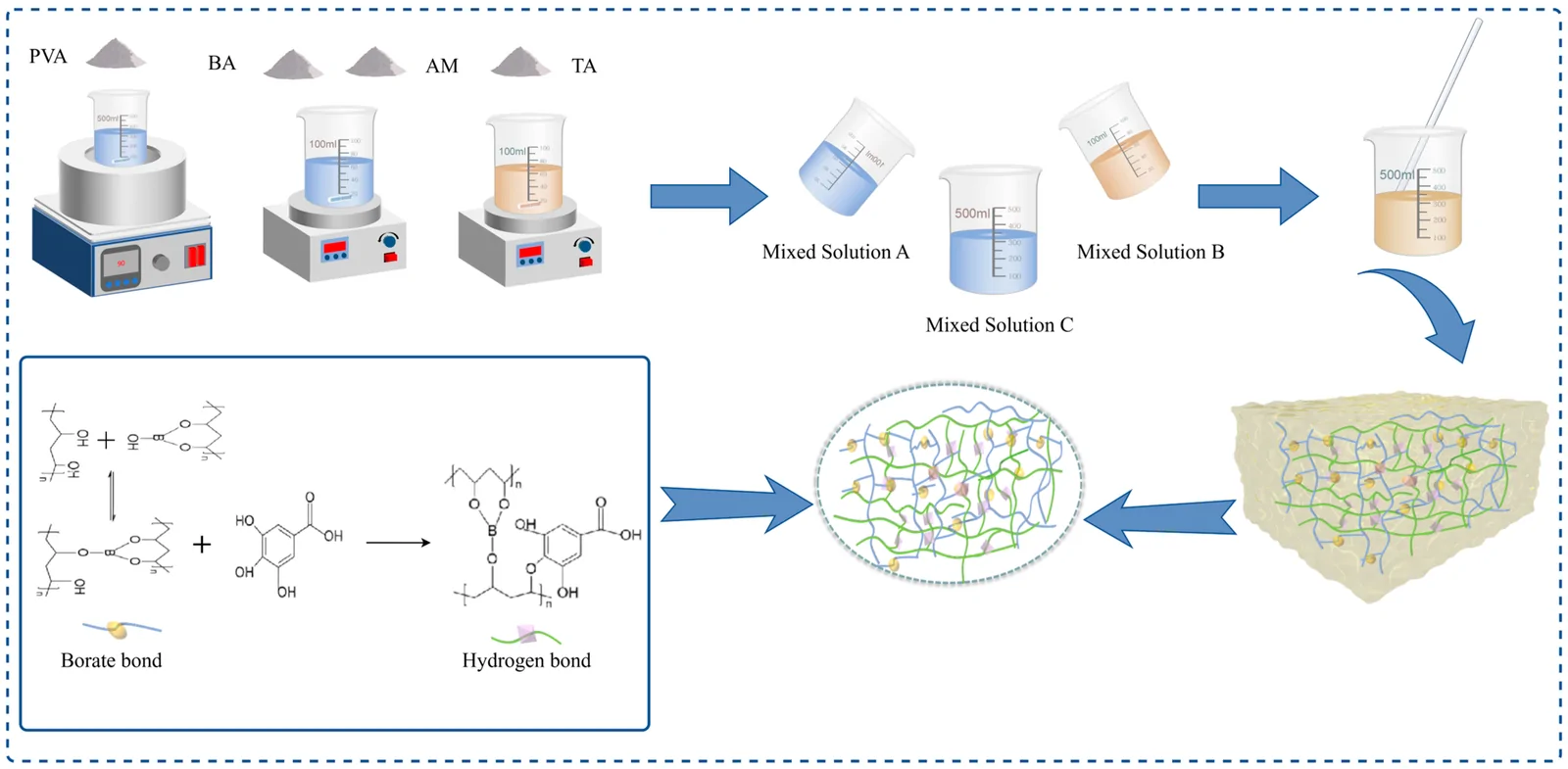
Synthesis route design.

Based on the reaction scheme described above, we designed the synthetic route shown in Figure 16.

### 3.3. Gel Characterization

The self-healing gel was tested using a IRTracer-100 Infrared Spectrometer (Shimadzu Corporation, Tokyo, Japan) with a resolution of 4 cm^−1^ and a wavenumber range of 4000–600 cm^−1^; the thermal stability of the self-healing gel was tested in the range of 30–800 °C using a TGA-2 Thermogravimetric Analyzer (Mettler-Toledo, Shanghai, China) under a nitrogen atmosphere at a heating rate of 10 °C/min; X-ray photoelectron spectroscopy (XPS) was performed to analyze the elemental composition, with a focus on the C1s, B1s, and O1s spectra.

### 3.4. Preparation of a 4% Water-Based Bentonite Slurry

During the preparation of the base slurry and drilling fluid, relevant standards established by the American Petroleum Institute (API) were followed. The method for preparing the prehydrated bentonite base slurry was as follows: 40 g of bentonite and 2 g of anhydrous sodium carbonate were added to 1000 mL of water and stirred continuously at a speed of 800 r/min for 16 h. In this study, the pre-hydrated bentonite base slurry was used directly to evaluate the gel’s loss-reduction performance; no other drilling fluid additives were added. A gel of a specific concentration was slowly added to the base slurry while stirring continued.

### 3.5. Study on the Compatibility of Base Pastes

In accordance with API standards, the API filtration loss of the drilling fluid was tested using a medium-pressure filtration loss tester (Model SD-4, Qingdao Haitongda Special Instrument Co., Ltd., Qingdao, China). The drilling fluid was placed in a water bath and heated to temperatures of 25, 55, and 80 °C for treatment. API filtration loss tests were then conducted at each of these temperatures.

A six-speed rotational viscometer (Model ZNN-D6, Qingdao Haitongda Special Instrument Co., Ltd., Qingdao, China) was used to test the rheological properties of the fluid. Rheological parameters such as apparent viscosity (AV), plastic viscosity (PV), and yield point (YP) were calculated using the following formulas based on the readings at 600 rpm (θ_600_) and 300 rpm (θ_300_):

AV = 1/2θ_600_ (1)

PV = θ_600_ − θ_300_ (2)

YP = 1/2(θ_300_ − PV) (3)

where AV is apparent viscosity (mPa·s); PV is plastic viscosity (mPa·s); YP is yield point (Pa).

### 3.6. Wetting Performance Test

Prepare 5%, 7%, and 10% gel solutions, apply them evenly to glass slides, and dry them in a vacuum oven at 60 °C until constant weight is reached. Using a contact angle measuring instrument, dispense 5.0 μL droplets of deionized water onto the surfaces of the different samples via a micropipette. After allowing the samples to stand for 2 min, the morphology of the gel surface and the water droplets was examined under magnification using the measurement software. Images of the water droplets were captured using the instrument’s built-in camera under illumination, and the contact angle values were measured and recorded.

### 3.7. Evaluation of High-Temperature, High-Pressure Plugging Performance

To evaluate the effect of dynamic self-healing gel particles on high-temperature, high-pressure plugging performance, two comparative formulations were prepared. Sample solution 1 contained walnut shell, fibres and mica particles, but no self-healing gel, and served as a benchmark representing traditional rigid particle/fibre bridging. Sample solution 2 contained the same matrix slurry and the same concentration of traditional materials, with the addition of 5 wt% self-healing gel particles.

(1) Take a measured quantity of base paste, weigh out the corresponding mass of treatment agents for each formulation according to the specified ratios, and prepare a solution of a specific concentration as Sample Solution 1 (Add 2% 10–20 mesh walnut shells, 1.5% 3–6 mm fibers, and 1% 10–20 mesh mica particles to 300 mL of 4% aged base paste.)

(2) Take a measured amount of base paste, weigh out the corresponding mass of treatment agent for each formulation according to the specified ratio, and prepare a gel particle solution of a specific concentration as Sample Solution 2 (the prepared gel was dried at 60 °C for 48 h, ground, and sieved to obtain 10–20 mesh gel particles; add 5% by weight of the 10–20 mesh gel particles to 300 mL of 4% aged base paste, followed by 2% by weight of 10–20 mesh walnut shells, 1.5% by weight of 3–6 mm fibers, and 1% by weight of 10–20 mesh mica particles.)

Once the mixture is thoroughly stirred and has fully absorbed water and expanded, pour it into the high-temperature, high-pressure static leak-sealing tester, install a steel mold with a 1 mm aperture, and set the output pressure to 1.4 MPa. After reaching the required test temperature, pressurize the system to 7 MPa, record the leakage volume of the sealing slurry every 10 min, and calculate the cumulative leakage volume. Adjust the pressure regulator on the nitrogen cylinder to increase the pressure, and conduct pressure-bearing leak-sealing tests at 10 MPa, respectively. After the tests are complete, remove the crack modules and observe the sealing effectiveness for each.

### 3.8. Study on Self Healing and Energy Consumption Mechanism of Gel

(1) Place the hydrogel in a 20 mm × 5 mm dumbbell-shaped mold and slowly apply contact pressure to obtain two dumbbell-shaped specimens. Stain one of the two dumbbell-shaped hydrogels with methyl blue. Then, cut both hydrogels in half lengthwise, ensuring that the cut surfaces of the differently colored hydrogels remain in contact. Add a drop of deionized water to the cut surface to moisten the cross-section, remove air from the interface, and provide the necessary medium for the cross-interface diffusion of macromolecule chains and the rebonding of functional groups. Observe the healing process of the cut surface in a sealed, humidified Petri dish at 25 °C.

(2) The microstructure of the self-healing hydrogel samples was examined using a scanning electron microscope (SEM, TESCAN GROUP, a.s., Brno, Czech Republic) To preserve the original microstructure of the samples, the hydrogel samples were freeze-dried after being immersed in liquid nitrogen. The dried samples were gold-sputtered before SEM imaging.

(3) Gels prepared using different methods were placed in a 20 mm × 5 mm dumbbell-shaped mold, and contact pressure was slowly applied to produce two dumbbell-shaped specimens. At room temperature, the mechanical properties of the gel specimens were tested using an electronic universal testing machine (Xiamen Yishite Instrument Co., Ltd., Xiamen, China). The tensile rate for the tensile test was 100 mm·min^−1^. The tensile rate for the cyclic tensile test was also 100 mm·min^−1^.

### 3.9. Research on Rheology

All rheological measurements were conducted using a complex fluid flow property tester (Haaker, Karlsruhe, Germany) equipped with a 25 mm parallel plate geometry and a 1000 μm gap.

The stress scanning experiments were conducted at an experimental temperature of 25 °C, with a fixed scan frequency of 1 Hz and a stress-scan range set from 0.01 to 100 Pa. By observing the dynamic response characteristics of the storage modulus (G’) and loss modulus (G”) under shear stress, the linear viscoelastic region of the gel was determined, and based on this, a fixed stress value within the linear region was selected for subsequent frequency scanning.

In the dynamic frequency scanning experiment, the gel is subjected to an angular frequency scan while ensuring that the applied stress is strictly controlled within the linear viscoelastic region. The test range is set from 0.1 to 100 rad/s, and the system continuously acquires and records the curves of G’ and G” as a function of angular frequency.

In the step strain test, the strain is set to 400% at a fixed frequency of 10 rad·s^−1^, held for 3 s, then the strain is set to 0.1% and held for 3 s; this sequence is repeated alternately three times.

## 4. Conclusions

To address the challenge of simultaneously achieving the required mechanical strength, self-healing capability, and pressure-bearing capacity for wellbore plugging in extreme downhole environments such as high temperature and high pressure, this study used polyvinyl alcohol (PVA) and boric acid (BA) as the matrix, and by introducing tannic acid (TA) and acrylamide (AM), this study developed a self-healing gel plugging material through dynamic covalent crosslinking, which utilizes a synergistic mechanism of borate dynamic covalent bonds and multiple hydrogen bonds. The study clarified the comprehensive performance of the self-healing gel plugging agent and elucidated its plugging mechanism. The conclusions are as follows:

(1) The characteristic B–O–C peak at 1080.2 cm^−1^ in FT-IR and the (B–O) peak at 191.94 eV and the (C–O) peak at 286.45 eV in XPS support the formation of dynamic covalent borate bonds and hydrogen bonds, resulting in a structure primarily based on borate bonds and reinforced by physical crosslinking via hydrogen bonds. TGA analysis shows that thermal degradation begins at 380 °C, with the residual mass remaining constant after 600 °C, indicating that the gel possesses structural stability in deep geothermal environments.

(2) Performance test results indicate that the gel exhibits excellent overall performance. In terms of rheological properties, under specific temperature conditions, as the gel concentration increased from 0% to 5%, the apparent viscosity at 25 °C rose from 11.25 mPa·s to 24 mPa·s, the yield point increased from 4.75 Pa to 16 Pa, and the API filtrate loss decreased from 23.0 mL to 17.2 mL; at 55 °C and a 5% gel concentration, the apparent viscosity (AV) and yield point (YP) reached 35 mPa·s and 28 Pa, respectively, indicating that the dynamic borate bonds maintain structural integrity even at high temperatures. The gel is compatible with drilling fluid and can effectively improve cuttings carrying capacity and mud cake density. In wettability tests, the 7% concentration gel exhibited an average contact angle of 71.30°, indicating a weakly hydrophilic surface.

(3) The patterns of gel formation and sealing, along with the self-healing mechanism, further elucidate the mechanism by which the gel remains in fractured formations. The gel follows the pattern of “particle migration and accumulation—in-situ crosslinking and gelation—overall gel pressure resistance.” In high-temperature, high-pressure tests conducted in a 1 mm crack, the 5% self-healing gel penetrated the pores through shear thinning and accumulated there. Through synergistic crosslinking via dynamic borate covalent bonds and hydrogen bonds, it formed a continuous sealing layer capable of withstanding a pressure of 10 MPa. This self-healing gel, which relies on the hydrolysis and reorganization of borate ester bonds combined with multiple hydrogen bonds, can fully heal an incision within 4 h. Furthermore, cyclic tensile testing showed that the gel with all components exhibited a stress of approximately 37 kPa at 200% strain, whereas the absence of AM or TA reduced this stress to 5 kPa and even lower, respectively, indicating that these two components form a synergistic energy-dissipation mechanism through enhanced hydrogen-bond physical crosslinking and borate ester bonds. Rheological analysis confirmed that the gel remains in a gel state at 180 °C, demonstrating its potential for high-temperature sealing.

In summary, the PVA/BA crosslinked self-healing gel developed in this study demonstrates good versatility and compatibility in water-based drilling fluid systems. Through synergistic crosslinking via dynamic borate covalent bonds and multiple hydrogen bonds, the physical crosslinking density—reinforced by tannic acid and acrylamide—endows the material with thermal stability and reduced hydrophilicity, thereby enhancing its sealing and self-healing capabilities under fracturing conditions. This self-healing gel technology provides an innovative solution for leak control and holds promising prospects for application in real-time wellbore leak control.

## Figures and Tables

**Table 1:** Contact angles as a function of gel concentration.

Gel Concentration	Average Contact Angle	Maximum Average Contact Angle
5%	62.23°	71.05°
7%	71.30°	73.60°
10%	63.40°	65.40°

**Table 2:** Pressure-Sealing Performance of Self-Healing Gel Particles.

No.	Total Time/Min	Stable Pressure/MPa	Total Leakage Volume/mL
Sample Solution 1	10	7.5	115
	20	9	115
Sample Solution 2	10	8	5
	20	9	50
	30	10	60
	40	10	60

## Data Availability

The data presented in this study are available on request from the corresponding authors. The data are not publicly available due to confidentiality and privacy restrictions.

## Abbreviations

The following abbreviations are used in this manuscript:

FT-IR	Fourier Transform Infrared Spectroscopy
XPS	X-ray Photoelectron Spectroscopy
AV	Apparent viscosity
PV	Plastic viscosity
YP	Yield point
SEM	Scanning electron microscope
API	American Petroleum Institute
TGA	Thermogravimetric analysis
AR	Analytical reagent
AM	Acrylamide
PVA	Polyvinyl alcohol
TA	Tannic acid
BA	Boric acid
PTFE	Polytetrafluoroethylene
